# NF-κB/p52 augments ETS1 binding genome-wide to promote glioma progression

**DOI:** 10.1038/s42003-023-04821-2

**Published:** 2023-04-22

**Authors:** Nicholas Sim, Yinghui Li

**Affiliations:** 1 School of Biological Sciences (SBS), Nanyang Technological University (NTU), 60 Nanyang Drive, Singapore, 637551 Singapore; 2 Institute of Molecular and Cell Biology (IMCB), A*STAR, Singapore, 138673 Singapore

**Keywords:** CNS cancer, Cancer genomics

## Abstract

Gliomas are highly invasive and chemoresistant cancers, making them challenging to treat. Chronic inflammation is a key driver of glioma progression as it promotes aberrant activation of inflammatory pathways such as NF-κB signalling, which drives cancer cell invasion and angiogenesis. NF-κB factors typically dimerise with its own family members, but emerging evidence of their promiscuous interactions with other oncogenic factors has been reported to promote transcription of new target genes and function. Here, we show that non-canonical NF-κB activation directly regulates p52 at the *ETS1* promoter, activating its expression. This impacts the genomic and transcriptional landscape of ETS1 in a glioma-specific manner. We further show that enhanced non-canonical NF-κB signalling promotes the co-localisation of p52 and ETS1, resulting in transcriptional activation of non-κB and/or non-ETS glioma-promoting genes. We conclude that p52-induced ETS1 overexpression in glioma cells remodels the genome-wide regulatory network of p52 and ETS1 to transcriptionally drive cancer progression.

## Abstract (web summary)

p52-induced ETS1 overexpression in glioma cells remodels the genome-wide regulatory network of p52 and ETS1 to transcriptionally drive cancer progression.

## Introduction

Gliomas are one of the deadliest cancers and account for roughly 80% of all central nervous system malignancies^1^. A well-known driver and hallmark of glioma progression is chronic inflammation, which manifests through the infiltration of tumour-associated macrophages, upregulation of inflammatory cytokines, angiogenesis and tissue remodelling^2,3^. As levels of inflammatory mediators such as TNFα and IL-1β are elevated, it causes the aberrant activation of the nuclear factor kappa-light-chain-enhancer of activated B cells (NF-κB), mitogen-activated protein kinase (MAPK)/extracellular-signal-regulated kinase (ERK) and signal transducer and activator of transcription 3 (STAT3) pathways which promote glioma progression^3,4^. The NF-κB signalling pathway consists of the canonical, non-canonical, atypical and DNA damage pathways and is essential for the regulation of multiple immune, inflammatory and developmental responses in normal tissues^5^. When hyperactivated, it can promote the development and progression of various cancers, including gliomas^6^, breast cancers^7^ and lymphomas^8^. Among these pathways, the canonical and non-canonical signalling arms are the most well-studied. Canonical NF-κB signalling can be activated through various cytokines, growth factors or pattern-recognition receptors^9^. Upon activation, the IκB kinase (IKK) complex is phosphorylated, which in turn phosphorylates IκBα, signalling it for ubiquitin-dependent proteasomal degradation. This results in the nuclear translocation of p50/p65 dimers which can either activate or suppress gene expression in a stimulus- and cell-context-dependent manner^10^. Conversely, non-canonical NF-κB signalling is triggered by a different subset of ligands such as B-cell activating factor (BAFF), receptor activator of nuclear factor kappa-Β ligand (RANKL) and tumour necrosis factor-like weak inducer of apoptosis (TWEAK)^5,6^. This promotes the stabilisation of NF-κB-inducing kinase (NIK), which activates IKKα and thereby leads to the proteolytical processing of p100 into p52^5^. The resultant mature p52 then translocates into the nucleus as p52/RelB dimers to exert transcriptional regulation.

Another key mediator of inflammation is the E26 (ETS) family of transcription factors (TFs), which are transcriptionally regulated downstream of MAPK signalling^11^. ETS TFs are involved in a wide range of biological processes, which include cell cycle control, cell proliferation, angiogenesis, tissue remodelling and differentiation^12,13^. ETS factors such as ETV2 and PU.1 have been reported to be overexpressed in gliomas, and their dysregulation promotes tumorigenesis and metastasis^14^.

Traditionally, NF-κB subunits form specific homo- or hetero-dimers among themselves to exert diverse transcriptional functions. However, various TFs such as STAT3, p53 and ERG have also been demonstrated to associate with NF-κB to repress or enhance the transcriptional activation of κB-dependent genes in human cancers^15–17^. Notably, NF-κB/p52 has been shown to physically interact and cooperate with ETS1 to regulate telomerase reactivation via the mutant telomerase reverse transcriptase (*TERT)* promoter, which is a cancer driver gene in almost 90% of human cancers^6,18^. However, the genome-wide regulatory interplay between p52 and ETS1 has yet to be further explored. Here, we show that p52 activation directly regulates *ETS1* expression through p52 binding at its promoter. The p52-driven overexpression of ETS1 resulted in the alteration of its genome-wide binding dynamics, which induced a change in the enrichment of its associated motifs and target genes. We further demonstrate that ETS1 functionally cooperates with p52 to promote glioma invasion and cell proliferation. Altogether, our data indicate the crucial role of NF-κB/p52 activation in enhancing the genomic binding landscape of ETS1 to drive glioma progression.

## Results

### NF-κB/p52 activation alters the genomic binding landscape of ETS1

**Fig. 1:**
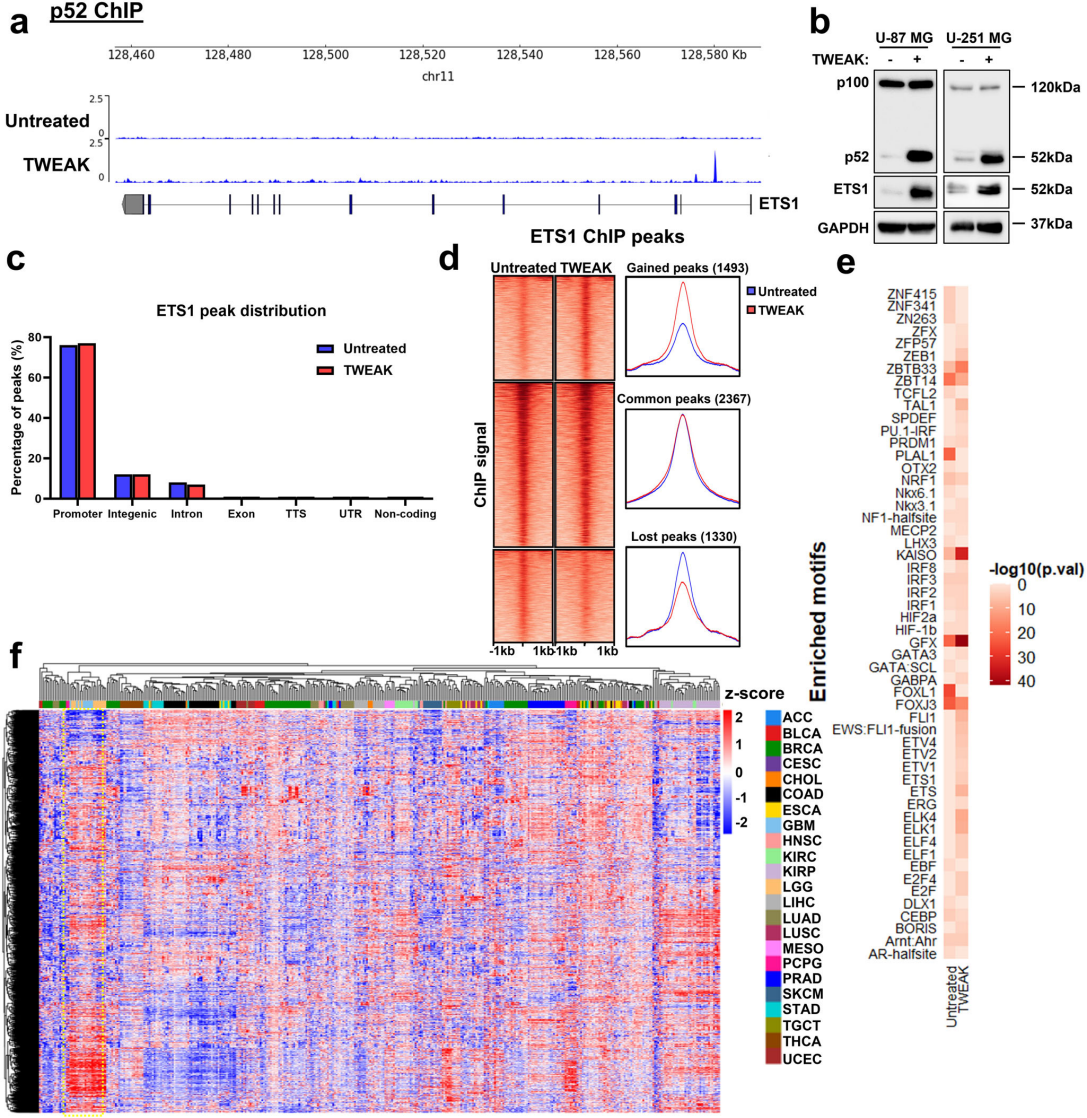
p52 activation remodels ETS1 DNA binding dynamics in glioma cells. **a** Genome browser view of *ETS1* gene locus and p52 ChIP-seq tracks in untreated and TWEAK-treated U-87 MG cells. Following TWEAK activation, p52 binds to the *ETS1* promoter. **b** ETS1 protein expression analysed by western blotting when NF-κB/p52 is activated via TWEAK exposure in glioma cell lines. **c** Genomic distribution of ETS1 DNA binding peaks in untreated and TWEAK-treated U-87 MG cells. **d** DNA binding dynamics of ETS1 in untreated and TWEAK-treated U-87 MG cells. Signal intensity across each region (±1 kb) is represented as a heatmap, and the average log2 fold change in signal of gained, lost or common ETS1 peaks following TWEAK treatment is displayed at the right. **e** Heatmap representation of the enriched ETS1 motifs in U-87 MG cells with and without TWEAK-induced NF-κB/p52 activation. **f** Heatmap depicting the chromatin accessibility signal at ETS1 regions following TWEAK activation in TCGA Pan-cancer ATAC-seq dataset. ATAC-seq signals of glioma patient samples (LGG—Brain Lower Grade Glioma, GBM—Glioblastoma multiforme) are marked in the yellow dotted box. TCGA study abbreviations can be found at https://gdc.cancer.gov/resources-tcga-users/tcga-code-tables/tcga-study-abbreviations.

The non-canonical NF-κB pathway has been documented to play critical roles in the progression of various malignancies and can be activated in response to a variety of ligands, including TWEAK^6,19–21^. To investigate the role of NF-κB/p52 in regulating the genomic occupancy of ETS1 in gliomas, we performed chromatin immunoprecipitation (ChIP) sequencing (ChIP-seq) using p52 and ETS1 antibodies in the glioma cell line, U-87 MG, with and without TWEAK treatment. There were 270 and 3697 unique p52 and ETS1 binding sites obtained, respectively, in untreated cells. Following TWEAK treatment, 11503 p52 and 3852 ETS1 binding sites were identified. (Supplementary Fig. 1). Interestingly, we detected the enrichment of p52 at the *ETS1* promoter upon TWEAK stimulation (Fig. 1a). Consequently, TWEAK-induced p52 activation resulted in the upregulation of ETS1 expression (Fig. 1b, Supplementary Fig. 6). We further confirmed this effect through CRISPR-mediated knockdown (KD) of *NFKB2* which inhibited TWEAK-induced upregulation of ETS1 (Supplementary Figs. 2 and 9).

Although p52 activation elevated ETS1 levels, it had little effect on both the number and distribution of ETS1 ChIP-seq peaks (Supplementary Fig. 1, Fig. 1c). However, we observed a distinct shift in the genomic binding landscape of ETS1 following TWEAK treatment (Fig. 1d). Further analysis of these differential sites revealed the increased enrichment of motifs for other ETS TFs such as FLI1, ELK4 and ELK1 (Fig. 1e), which have been implicated in cancer progression, following TWEAK-induced p52 activation^22–24^. These TFs were also among the most highly expressed ETS factors in glioma patients (Supplementary Fig. 3). We also observed the enrichment of motifs for non-ETS factors, including KAISO and ZEB1, that have been documented to mediate the tumorigenic progression of gliomas^25,26^. This suggested that the p52-driven augmentation of ETS1 genomic binding may increase the co-association and cooperativity between ETS1 and different transcriptional co-activators that are overexpressed in gliomas. These changes in the ETS1 DNA binding dynamics may potentially enhance the transcription of various new target genes, which can promote cancer progression.

To gain molecular insights into the relevance of the TWEAK-induced ETS1 genomic profiles in gene regulation, we interrogated the TCGA pan-cancer ATAC-seq dataset^27^. Chromatin accessibility promotes TF occupancy and the activity of DNA regulatory elements, which is crucial for gene transcription. When dysregulated in cancers, it can result in the overexpression of oncogenes through the remodelling of enhancer signatures^28^, pioneer factor-driven de novo enhancer formation^29^ and/or chromosomal topology disruption^30^. Among the 23 cancers within the TCGA ATAC-seq dataset, we found that the regions of accessible DNA which overlapped p52-activated ETS1 binding sites were highly enriched and specific to glioma patients (Fig. 1f). Collectively, these findings demonstrate that p52 activation drives a glioma-specific alteration in the genomic landscape of ETS1 that is enriched in accessible chromatin. Such DNA binding dynamics can promote the co-association of ETS1 with other oncogenic TFs to drive cancer-specific gene transcription.

### p52-activated ETS1 alters the transcriptomic landscape in glioma

**Fig. 2:**
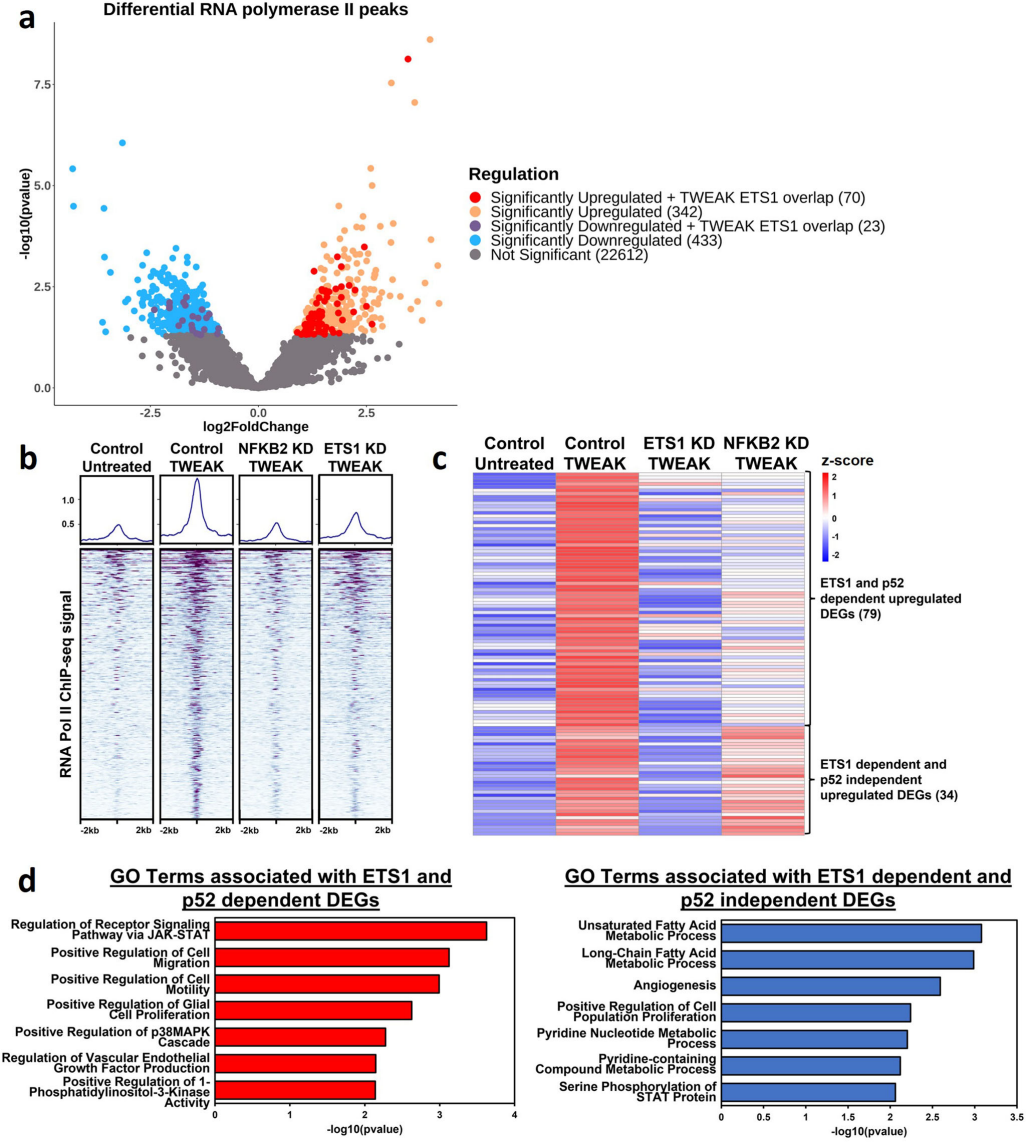
p52-driven ETS1 alters RNA polymerase II recruitment and the transcriptomic landscape of glioma cells. **a** Volcano plot depicting differential RNA polymerase II binding following NF-κB/p52 activation and those bound by ETS1. **b** DNA binding heatmap of the significantly upregulated RNA polymerase II sites following NF-κB/p52 activation and the effect of *NFKB2* and *ETS1* KD. **c** Heatmap depicting ETS1-dependent, significantly upregulated genes following NF-κB/p52 activation. **d** Bar chart depicting enriched GO terms from significantly upregulated ETS1 and p52-dependent differentially expressed genes (DEGs) as well as ETS1-dependent and p52-independent DEGs.

To further examine the transcriptional activities driven by TWEAK-induced p52 activation in gliomas, we performed ChIP-seq using RNA polymerase II (RNA Pol II) antibody in U-87 MG cells treated with and without TWEAK. Differential binding analysis revealed that p52 activation alters the recruitment of RNA Pol II genome-wide, leading to its significant upregulation at 412 sites and significant downregulation at 456 regions. Interestingly, we find that ETS1 is co-localised in approximately 20% of all upregulated RNA Pol II sites (Fig. 2a). We further verified that the p52-driven ETS1 binding dynamics is crucial for the maintenance of these upregulated RNA Pol II regions as TWEAK-driven RNA Pol II recruitment was abolished with CRISPR/Cas9-mediated knockdown of *NFKB2* and *ETS1* expression (Fig. 2b). These results demonstrate that the p52-induced ETS1 genomic landscape regulates the recruitment of the transcriptional machinery in glioma cells.

Following this, we performed RNA-seq to profile the target genes that are transcriptionally regulated by p52-activated ETS1. This revealed that TWEAK treatment led to the upregulation of 113 ETS1-dependent differentially expressed genes (DEGs). Among them, 79 were also dependent on p52, indicating that these genes are driven by p52-induced ETS1, while 34 were upregulated independent of p52 (Fig. 2c). Next, we performed gene ontological analysis on these two groups of upregulated DEGs. This analysis showed that both groups of DEGs shared enrichment of similar biological processes relating to cell proliferation and JAK-STAT pathway activation. Intriguingly, the ETS1 and p52-dependent DEGs were specifically enriched for terms associated with cell migration, VEGF production and MAPK activation (Fig. 2d), which drive glioma development and progression^31–33^. Taken together, these findings demonstrate the role of p52-induced ETS1 genomic binding in the transcriptional activation of distinct target genes that are critical for glioma progression.

### p52-induced ETS1 expression drives glioma invasion and cell proliferation

**Fig. 3:**
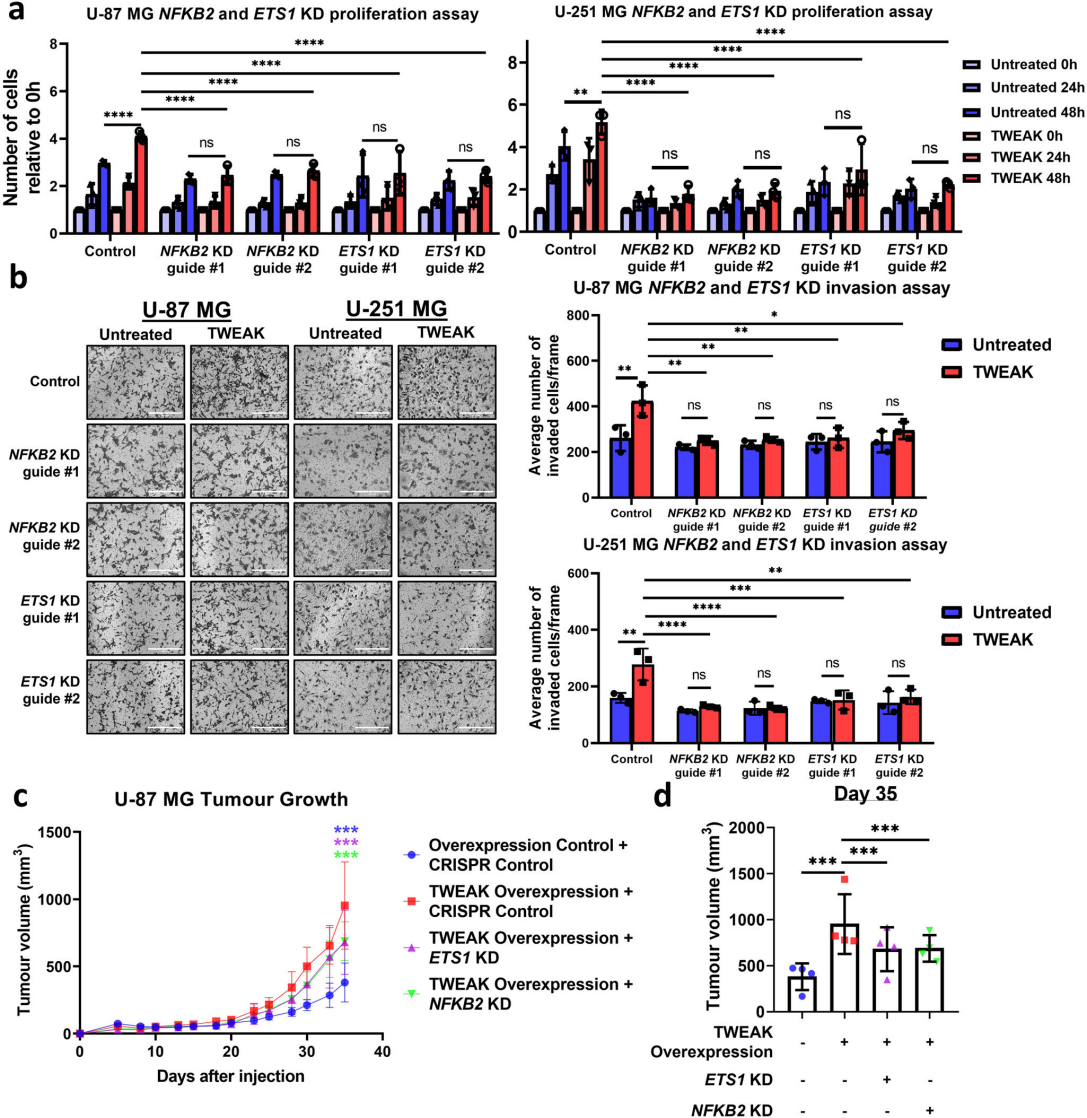
p52 drives the invasion and proliferation of glioma cells through ETS1. **a** Proliferation assay plot depicts the average number of cells counted relative to 0 h (mean ± s.d) in U-87 MG and U-251 MG *NFKB2* and *ETS1* KD cells with and without TWEAK-induced NF-κB/p52 activation. The data shown represent *n* = 3 biological replicates. **b** Transwell invasion assay was performed in U-87 MG and U-251 MG *ETS1* KD cells with and without TWEAK treatment. Representative images from *n* = 3 biological replicates are shown. Scale bars: 400 µm. Representative plots depict the average number of invaded cells (mean ± s.d) across five fields. Representative plots depicting the average tumour volume (mean ± s.d) of U-87 MG cells injected into mice that express either CRISPR control, *NFKB2* KD or *ETS1* KD and overexpression control or TWEAK **c** over 35 days and **d** on day 35. The data shown represent *n* = 4 mice. Two-way ANOVA was used for all statistical analyses where *P < 0.05; **P < 0.01; ***P < 0.001.

To functionally validate the role of p52-activated ETS1 in glioma progression, we targeted *NFKB2* and *ETS1* expression in U-87 MG and U-251 MG cells using CRISPR-Cas9 gene editing tools and verified their downregulation through western blotting (Supplementary Figs. 2, 4, 9 and 10). Here, we found that TWEAK-induced non-canonical NF-κB activation resulted in the increased proliferation and invasion of both glioma cell lines (Fig. 3a, b). However, these phenotypes were abolished following the loss of NFKB2 or ETS1 expression (Fig. 3a, b). We further confirmed the p52-driven tumorigenic effects in vivo whereby TWEAK overexpression significantly increased the growth of U-87 MG tumour xenografts, but this was reduced by CRISPR/Cas9-mediated knockdown of NFKB2 or ETS1 (Fig. 3c, d, Supplementary Fig. 5). Taken together, these observations demonstrate that TWEAK-induced p52 activation promotes glioma progression through enhanced ETS1 expression and remodelling of its genomic landscape, which is highly enriched at the over-accessible chromatin of glioma patients.

### ETS1 is crucial for the maintenance of p52 DNA binding

**Fig. 4:**
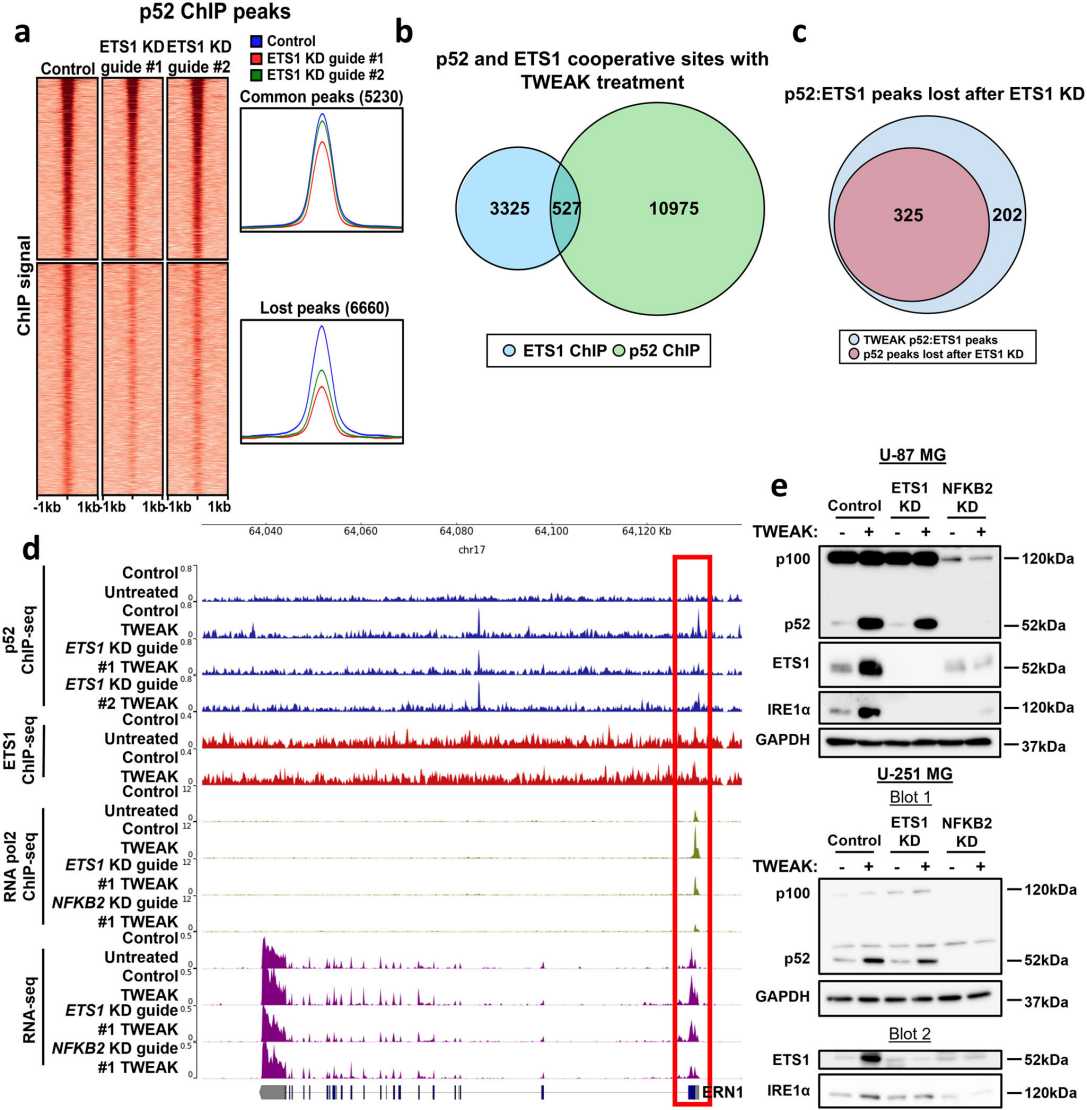
ETS1 regulates the genome-wide binding of p52. **a** DNA binding dynamics of p52 in TWEAK-treated U-87 MG cells following *ETS1* KD. Venn diagram depicting **b** the number of cooperative sites between p52 and ETS1, and **c** the number of p52:ETS1 co-binding sites lost following *ETS1* KD. **d** RNA-seq tracks and p52, ETS1 and RNA pol II ChIP-seq tracks at the *ERN1* promoter following NF-κB/p52 activation as well as *NFKB2* and *ETS1* KD. Genomic changes observed at the *ERN1* promoter are marked in a red box. GRCh38 assembly was used. **e** p100/p52, ETS1 and IRE1α protein expression analysed by western blotting in U-87 MG and U-251 MG cell lines following NF-κB/p52 activation as well as *NFKB2* and *ETS1* KD. The western blot involving U-251 MG cells was run over two blots at the same time and loaded with the same amount of protein. The proteins probed on each blot are indicated under Blot 1 and Blot 2.

ETS1 can physically interact with various TFs such as Runx1, Pit-1 and HIF-2α where the DNA binding affinity of ETS1 or its binding partner is modulated^34–36^. As ETS1 also physically co-associates and cooperates with p52 in gliomas^6,18^, we sought to examine the role of p52-activated ETS1 expression in the genomic binding of p52. To investigate this, p52 ChIP-seq was performed on CRISPR control and *ETS1* KD U-87 MG cells following TWEAK-induced non-canonical NF-κB activation. In the absence of ETS1, we unexpectedly found that almost 60% of all p52 DNA binding sites were lost (Fig. 4a) while 34 binding sites were upregulated (too few sites to be effectively seen in the figure), indicating that ETS1 regulates genome-wide p52 binding. Furthermore, approximately 60% of the p52:ETS1 co-association sites were among the lost p52 sites (Fig. 4b, c), suggesting that p52 binds indirectly to the DNA through ETS1 at the genomic loci where both TFs cooperate. We also noted that these co-localised regions only constitute a subset of all lost p52 binding sites, implying that ETS1 can also indirectly regulate and dictate p52 DNA binding through alternate mechanism(s).

To validate the requirement of ETS1 in the maintenance of p52 binding at sites of cooperation, we selected the genomic locus of the *ERN1* gene, which encodes for Inositol-requiring transmembrane kinase/endoribonuclease 1α (IRE1α) as its promoter was bound by ETS1, independent of p52 activation. During TWEAK-induced non-canonical NF-κB activation, p52 binding was found to be enriched at the *IRE1α* promoter. This was also accompanied by increased RNA Pol II recruitment and transcriptional activity. In contrast, repression of ETS1 diminished p52 enrichment at the *ERN1* promoter and abolished TWEAK-driven RNA Pol II recruitment and gene transcription. We also noted a similar observation when NFKB2 expression was repressed, indicating that the p52:ETS1 cooperativity is required for the transcriptional activation of *IRE1α* (Fig. 4d). Further analysis also showed that the functional cooperativity between p52 and ETS1 following TWEAK treatment resulted in the upregulation IRE1α protein expression. Conversely, CRISPR-mediated downregulation of ETS1 or NFKB2 expression abolished the induction of IRE1α (Fig. 4e, Supplementary Fig. 7). Altogether, these results demonstrate the ability of ETS1 to dictate p52 genome-wide DNA binding through direct and indirect mechanisms. Furthermore, our data highlights the cooperative nature of p52 and ETS1, whereby the co-localisation of both TFs at gene promoters can enhance target gene expression.

### p52:ETS1 regulation of *IRE1α* drives glioma invasion and proliferation

**Fig. 5:**
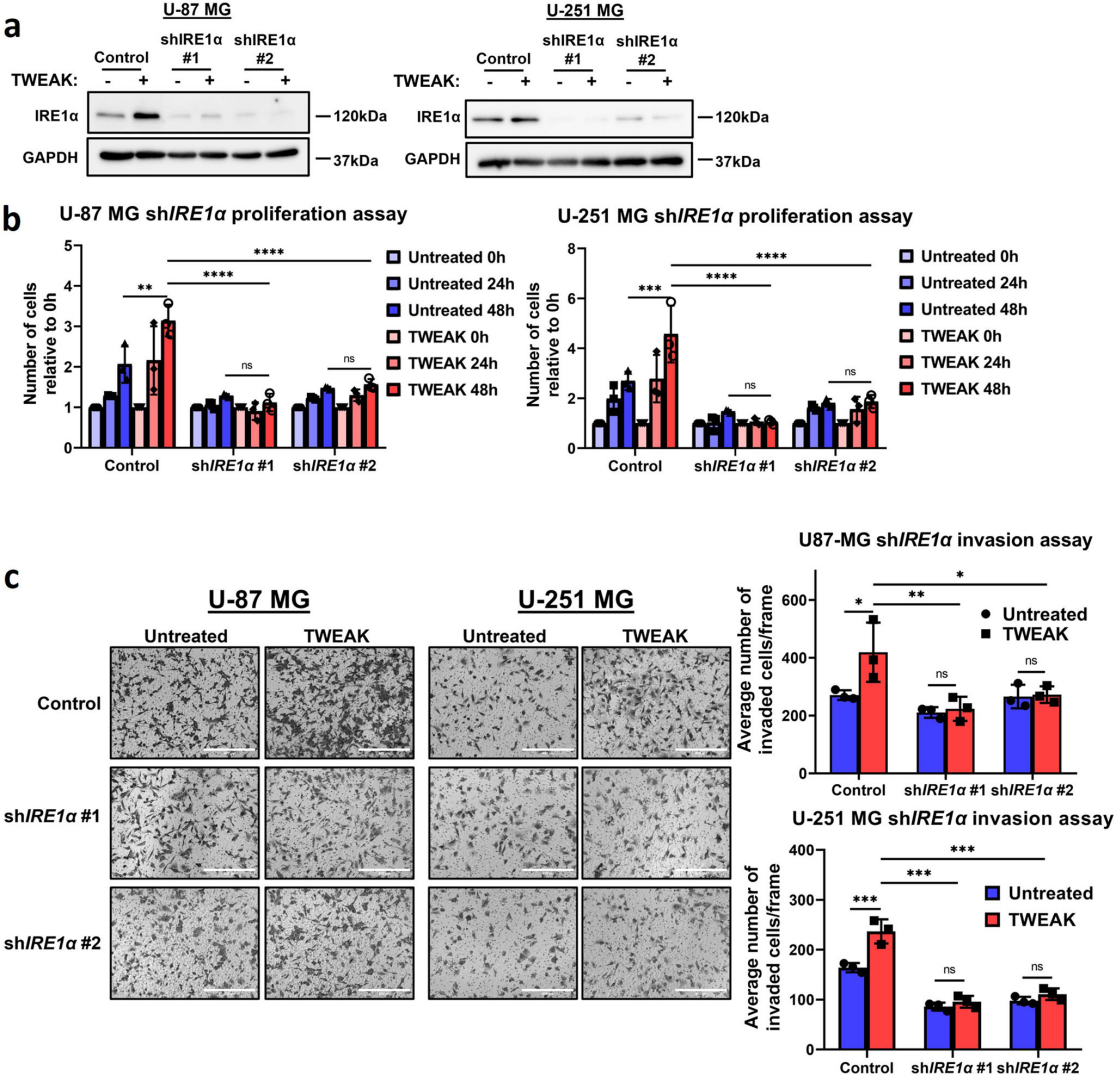
p52:ETS1 cooperativity promotes glioma cell invasion and proliferation through IRE1α. **a** IRE1α protein expression analysed by western blotting to validate shRNA-mediated repression of *IRE1α* in U-87 MG and U-251 MG cells. **b** Plot depicts the average number of cells relative to 0 h (mean ± s.d) in U-87 MG and U-251 MG sh*IRE1α*. **c** Transwell invasion assay was performed in U-87 MG and U-251 MG sh*IRE1α* cells. Representative images from *n* = 3 biological replicates are shown. Scale bars: 400 µm. Plots (right) depict the average number of invaded cells (mean ± s.d) across five fields. All functional experiments were compared in the presence and absence of TWEAK treatment. Two-way ANOVA was used for all statistical analyses. *P < 0.05; **P < 0.01; ***P < 0.001.

Having identified *IRE1α* as a target gene of p52 and ETS1 cooperativity, we proceeded to validate its functional roles in glioma progression by performing short hairpin RNA (shRNA)-mediated KD of the gene in U-87 MG and U-251 MG cell lines (Fig. 5a, Supplementary Fig. 8). Here, we observed that the loss of IRE1α expression resulted in the significant downregulation of glioma cell invasion and proliferation, which was not rescued by TWEAK treatment (Fig. 5b, c). These observations confirm the oncogenic role of IRE1α in glioma cells and suggest that p52:ETS1 driven *IRE1α* expression is crucial for glioma survival and progression. Our data further provides functional evidence for the relevance of p52:ETS1 cooperativity in the transcriptional regulation of glioma-promoting genes.

## Discussion

In this study, we uncovered *ETS1* as a target gene of the non-canonical NF-κB pathway. The roles of non-canonical NF-κB signalling and ETS1 have both been well documented to regulate glioma migration, invasion and cell proliferation^37,38^ Furthermore, p52 and ETS1 have been described to function cooperatively at the mutant *TERT* promoter to drive telomerase expression in gliomas^6,18^. These studies implicate the critical role of p52:ETS1 cooperativity in the transcriptional activation of cancer driver genes. Here, we provide evidence for the direct regulation of p52 at the *ETS1* promoter to activate its expression. The overexpression of ETS1 alters its genomic binding dynamics and transcriptional activities, which in turn impacts glioma invasion and survival.

On its own, ETS1 is expressed in an autoinhibited form^39^ and is unable to bind to DNA. But in several cancers, it has been shown to dimerise with other TFs, such as Pax5^40^, Runx1^36^ and Runx2^41^. This increases its DNA binding affinity, which can enhance the transcription of new target genes and potentially regulate oncogenic processes. Here, we report a similar signature whereby TWEAK-induced p52 activity resulted in the increased enrichment of oncogenic motifs, such as various ETS factors, KAISO and ZEB1, at ETS1 binding sites and augmented genome-wide ETS1 DNA binding, which altered the transcriptomic landscape of gliomas. We also observed that TWEAK-regulated ETS1 DNA binding was highly enriched at over-accessible chromatin regions in glioma patients, and ETS1 activation consequently supports glioma invasion and tumour proliferation. Altogether, these results highlight the functional importance and clinical relevance of p52-driven ETS1 genomic binding in glioma-specific progression.

As reports indicate that p52:ETS1 can cooperatively activate non-κB and/or non-ETS target genes^6,18^, we investigated this regulatory mechanism genome-wide. Unexpectedly, we found that nearly 60% of all p52 binding sites were lost following ETS1 KD in TWEAK-activated glioma cells. These data suggest that ETS1 is required to sustain p52 DNA binding. We further noted that among these lost p52 sites, a small proportion comprised the co-localised p52:ETS1 regions, while almost 95% of the lost p52 peaks did not interact with ETS1 (Fig. 4b, c). This implies that ETS1 primarily regulates the p52 genomic binding landscape through indirect mechanism(s). We have earlier suggested that at sites of cooperativity, p52 binds indirectly to DNA through ETS1. Similarly, NF-κB has been reported to interact with AP-1 TFs via recruitment without binding directly to DNA^42^. As AP-1 TFs are direct target genes of ETS1^43^, it is plausible that p52-activated ETS1 enhances AP-1 expression, which increases the availability of DNA-bound AP-1, thereby providing an anchor for p52 to establish indirect DNA binding.

To investigate the direct cooperation between p52 and ETS1, we focused on the genomic locus of *ERN1*. IRE1α is an enzyme associated with the unfolded protein response that is activated under endoplasmic reticulum stress, and its involvement in cancer progression has been documented in gliomas^44^, breast cancer^45^ and prostate cancer^46^. We showed that in the absence of non-canonical NF-κB signalling, binding of ETS1 to the *ERN1* promoter is insufficient to promote its transcriptional activation. But during TWEAK induction, p52 is enriched at the *ERN1* promoter, resulting in p52:ETS1 co-localisation and the recruitment of RNA Pol II. This leads to the upregulation of *IRE1α* expression, thereby promoting glioma progression. While our data highlight the prerequisite of ETS1 for sustaining p52 DNA binding and provide genomic evidence for the indirect binding of p52 to DNA through ETS1, this study further implicates the importance of TWEAK-regulated p52:ETS1 cooperativity in the transcriptional control of glioma-promoting genes.

Our study highlights a previously unrecognised role of non-canonical NF-κB activation in ETS1 regulation, whereby p52-induced ETS1 expression results in a multitude of genomic, transcriptional and functional events in glioma cancers. We further investigated the direct cooperative role of TWEAK-driven p52:ETS1 and demonstrated its capacity to regulate and drive glioma progression. Intriguingly, we found that NF-κB-driven ETS1 regulated p52 DNA binding predominantly through indirect means, highlighting an overall complex regulatory relationship between these two TFs. Hence, our results emphasise the relevance of non-canonical NF-κB activation and ETS1 in glioma progression, thereby highlighting the immense potential of targeting the p52-ETS1 regulatory axis in cancer therapy.

## Methods

### Cell lines and reagents

U-87 MG (ATCC) and U-251 MG (Sigma-Aldrich) (formerly known as U-373 MG) (ECACC 09063001) GBM cell lines were kindly gifted from Prof. Phillip Koeffler at the Cancer Science Institute, Singapore^47^. Cell lines were maintained in Dulbecco’s modified Eagle’s medium (DMEM; HyClone) supplemented with 10% fetal bovine serum (FBS; Sigma) and regularly tested negative for mycoplasma contamination using Mycoplasma PCR Detection Kit (Applied Biological Materials Inc; G238). Cell lines were not authenticated via STR profiling by us. Recombinant human TWEAK (PeproTech; 310-06) was reconstituted according to the manufacturer’s recommendations. All GBM cell lines were treated with 10 ng/ml of TWEAK.

### Plasmids

lentiCRISPRv2 (Addgene; Cat #52961) was used for generating ETS1 KD in U-87 MG and U-251 MG cell lines. sgRNAs targeting ETS1 and NFKB2 were designed on Benchling^48^, where sgRNAs with the highest On-Target score and Off-Target score were selected (Supplementary Table 1).

pLKO.TRC (Addgene; Cat#10878) was used for generating shIRE1α in U-87 MG and U-251 MG cell lines (Supplementary Table 1).

The TWEAK overexpression construct was generated by amplifying the soluble TWEAK (sTWEAK) sequence from cDNA (Supplementary Table 1) and replacing EGFP in pLJM1-EGFP (Addgene; 19319) with its puromycin resistance gene substituted with hygromycin resistance. Subsequently, pLJM1-sTWEAK was used as a template to amplify sTWEAK with a 3x Flag tag before being cloned into pLJM1-EGFP.

### Mouse subcutaneous xenograft

All animal experiments were performed in compliance with protocols approved by the Nanyang Technological University Institutional Animal Care and Use Committee (IACUC). Six- to ten-week-old male *Rag-/- IL2γ-/-* mice were housed at the animal facility of the Nanyang Technological University School of Biological Sciences.

When performing the subcutaneous xenograft, mice were anaesthetised with isofluorane and injected with 60 µl of 2.5 × 10^6^ U-87 MG cells in sterile PBS mixed with 33% Matrigel (Corning). The U-87 MG cells injected expressed the following combinations of plasmid (Supplementary Table 2). Primary tumours were measured for up to 5 weeks with callipers.

### Lentivirus production and transduction

For lentivirus production, 3 × 10^6^ HEK293T cells were plated per 10-cm plate. The next day, lentiCRISPR v2 or pLKO with pMDL, VSVG and REV were transfected into the cells via the calcium chloride method. The cells were washed after 8 h and replenished with DMEM supplemented with 10% FBS. After 24 h, the supernatant containing viral particles was collected.

For lentivirus transduction, virus and polybrene were added to GBM cells. Media was changed 24 h after transduction, and appropriate antibiotics were added 48 h after transduction.

### Cell proliferation assay

U-87 MG and U-251 MG cells were seeded at a density of 5 × 10^4^ and 1 × 10^4^ cells per well in a 12-well plate respectively. Cells were incubated overnight to allow attachment before being starved for 8 h in serum-free DMEM. After which, the number of cells was counted (this number will be referred to as 0h) and 10 ng/ml TWEAK was added. After 24 and 48 h, the number of cells was counted again.

### Invasion assay

Invasion assay was performed using 24-well Transwell inserts (Corning). Matrigel (Corning) was diluted to 200 µg/ml with Optimem (Gibco); 100 µl was added into each insert and incubated at 37 °C for 2 h. GBM cells were starved for 8 h beforehand; subsequently, the GBM cells were treated with 10 ng/ml TWEAK. After 24 h, the GBM cells were trypsinised, counted, resuspended in 100 µl Optimem containing 10 ng/ml TWEAK and seeded above the Matrigel layer. Then, 600 µl DMEM supplemented with 10% FBS was added to the lower chamber. The GBM cells were then incubated at 37 °C for 24 h. Following this, the transwell inserts were fixed in 70% ethanol and stained in 0.2% crystal violet, and non-invading cells on the top chamber were removed with cotton buds. Invading cells were then visualised with an inverted microscope at five different frames. The number of invading cells were counted in each frame and averaged.

### Western blot analysis

Western blotting was performed using standard methods, and the following antibodies were used for analysis: anti-NFkB2 (Cell Signalling; 3017), anti-ETS1 (Cell Signalling; 14069), anti-IRE1α (Santa Cruz; 390960) and anti-GAPDH (Santa Cruz; 32233). Antibody dilutions used were 1:10000 for anti-GAPDH and 1:1000 for the other antibodies.

### RNA library construction, sequencing and analysis

Total RNA was isolated using TRIzol Reagent (Invitrogen) according to the manufacturer’s protocol and then treated with TURBO DNA-free kit (Invitrogen; AM1907). RNA-seq library was constructed with NEBNext Ultra II Directional RNA Library Prep Kit for Illumina (NEB; E7765) through the polyA mRNA workflow. RNA-seq libraries were sequenced on HiSeqX. All RNA-seq experiments were performed with three biological replicates.

The quality of sequencing reads was assessed using FastQC^49^, and adaptors were removed with Trimmomatic^50^. RNA-seq reads were mapped to hg38 with STAR^51^ and processed by featureCounts^52^. Differential analysis was performed with DESeq2^53^, where differentially expressed genes were defined by having *P*-value ≤0.05 and fold change ≥1.5. Gene ontological analysis was performed using PANTHER^54,55^.

### Chromatin immunoprecipitation (ChIP), library construction, sequencing and analysis

Ten million U-87 MG cells were crosslinked with 1% formaldehyde (Sigma) for 10 min and quenched with 0.125 M glycine for 5 min at room temp. Cells were washed with PBS and then lysed with an SDS buffer supplemented with protease inhibitors (Roche). Lysed samples were then sonicated with Bioruptor Plus (Diagenode) to achieve DNA fragments between 200 and 500 bp. Chromatin was then precleared with blocked Protein G Sepharose beads (GE Healthcare) and incubated with the following antibodies: 2 µg anti-NFkB2 (Bethyl Laboratories; A300-BL7039), 4 µg anti-ETS1 (Active Motif; 39580) and 2 µg anti-RNA polymerase II clone CTD4H8 (EMD Millipore; 05-623). The beads were subsequently washed in the following order of buffers: SDS buffer, high salt buffer, lithium chloride wash buffer and Tris-EDTA buffer. After which, the ChIP DNA was de-crosslinked and eluted. The eluted DNA was used for library preparation with NEBNext Ultra II DNA Library Prep Kit (NEB; E7645) according to the manufacturer’s protocol.

ChIP DNA libraries were sequenced on HiSeqX. The quality of sequencing reads was assessed using FastQC^49^. Trimmomatic^50^ was used to remove adaptors and verified by running FastQC once more. Reads were mapped to hg38 using Bowtie2^56^, and peaks were called using MACS2 with subtraction of the input signal. Blacklisted regions were removed from the called peaks using bedtools^57^. The gained and lost ETS1 peaks were determined using bedtools. Differential RNA polymerase II peaks were analysed using DiffBind^58^.

Heatmaps showing signal distribution over ChIP peak regions were generated using deepTools^59^. ETS1 binding motifs were identified by HOMER^60^ with the hg38 genome as background and MEME-ChIP^61^.

### Integration with TCGA pan-cancer ATAC-seq data

Pan-cancer patient ATAC-seq sample counts^27^ from The Cancer Genome Atlas (TCGA) were retrieved using the UCSC Cancer Browser (http://xena.ucsc.edu/welcome-to-ucsc-xena/)^62^. Heatmaps were generated using R Statistical Programming Language and ggplot2^63^.

### Statistics and reproducibility

In all experiments, a two-way ANOVA statistical test was used. All in vitro proliferation and invasion assays were performed in three biological replicates. The in vivo assay was performed in four biological replicates. Asterisks represent the degree of statistical significance, *P < 0.05; **P < 0.01; ***P < 0.001. All statistical analyses and graphics were performed using GraphPad Prism software.

### Reporting summary

Further information on research design is available in the Nature Portfolio Reporting Summary linked to this article.

## Supplementary information

Supplementary Information

Description of Additional Supplementary Files

Supplementary Data 1

Reporting Summary

## Data Availability

Source data for Figs. 1c, 2a, 2d, 3, 5b, c can be found in Supplementary Data 1. The ChIP-seq and RNA-seq datasets generated during and/or analysed during the current study had been deposited in the GEO (Gene Expression Omnibus) database under the accession number GSE207982.
