# Supplementary material for: NF-κB/p52 augments ETS1 binding genome-wide to promote glioma progression
Source: Commun Biol. 2023 Apr 22;6:445. doi: 10.1038/s42003-023-04821-2 (PMC10122670; doi:10.1038/s42003-023-04821-2)
Supplement: Supplementary file 1 — Supplementary Information [file 42003_2023_4821_MOESM1_ESM.pdf]

Supplementary Figures

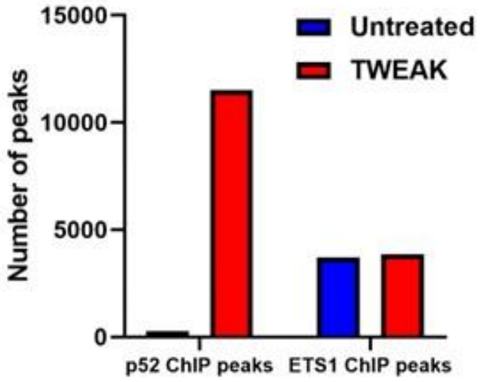

Supplementary Figure 1. Number of unique p52 and ETS1 ChIP peaks in both untreated and TWEAK treated U-87 MG cells.

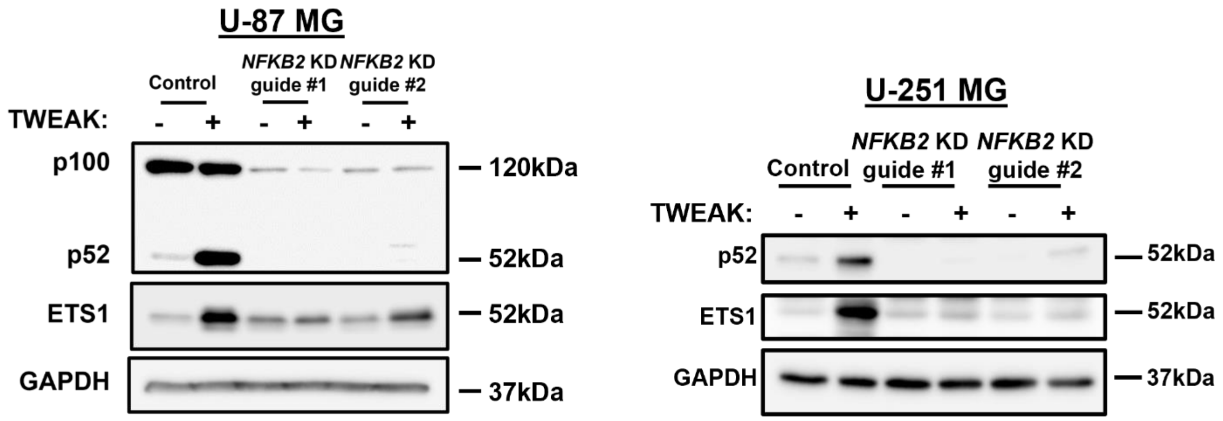

Supplementary Figure 2. p52 and ETS1 expression in U-87 MG and U-251 MG cells following *NFKB2* knockdown and TWEAK treatment analysed through western blotting.

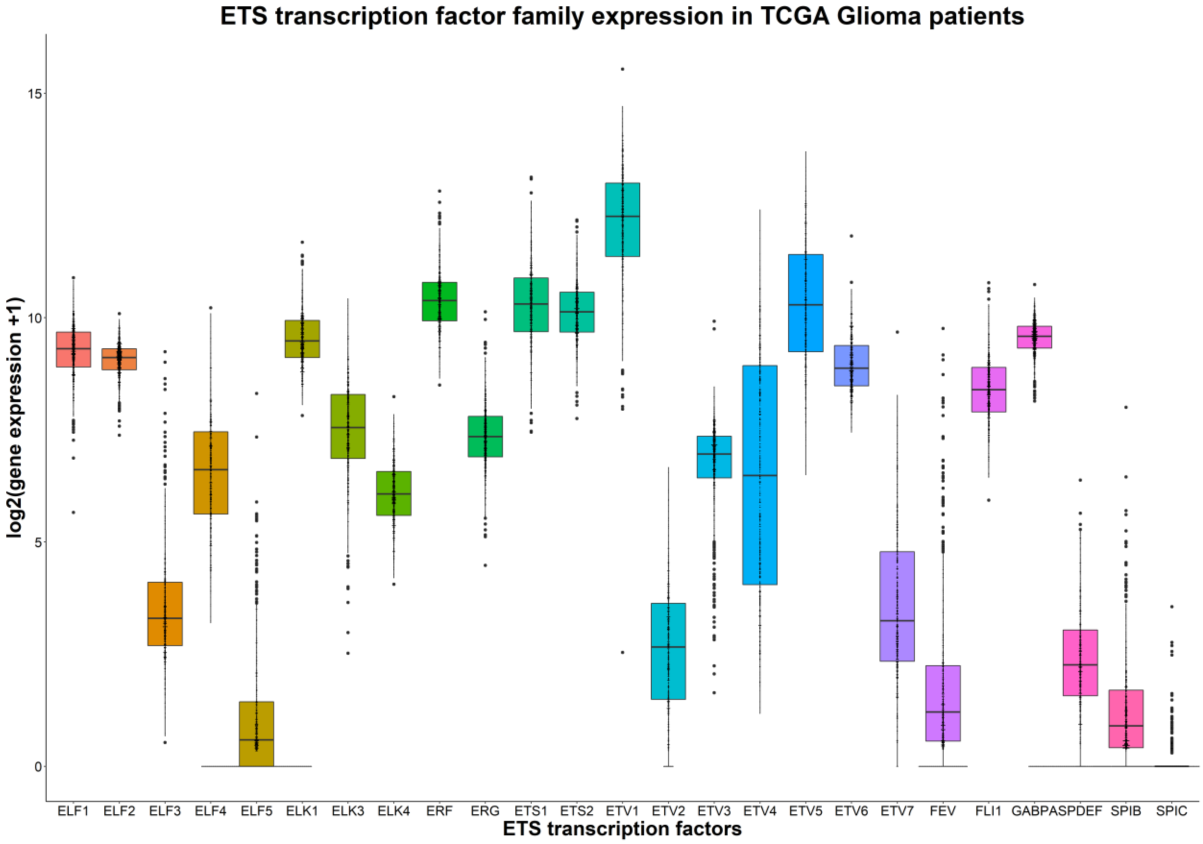

Supplementary Figure 3. Gene expression of ETS transcription factors in glioma patients (*n* = 702) from TCGA RNA-seq dataset. The box plots define the median, upper and lower quartiles.

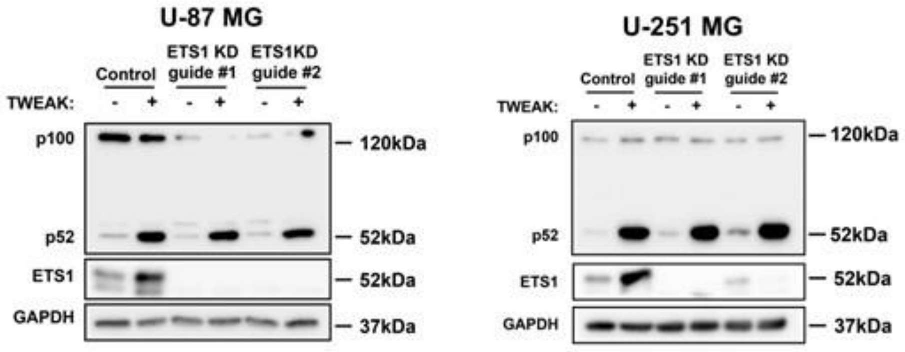

Supplementary Figure 4. p52 and ETS1 expression in U-87 MG and U-251 MG cells following *ETS1* knockdown and TWEAK treatment analysed through western blotting.

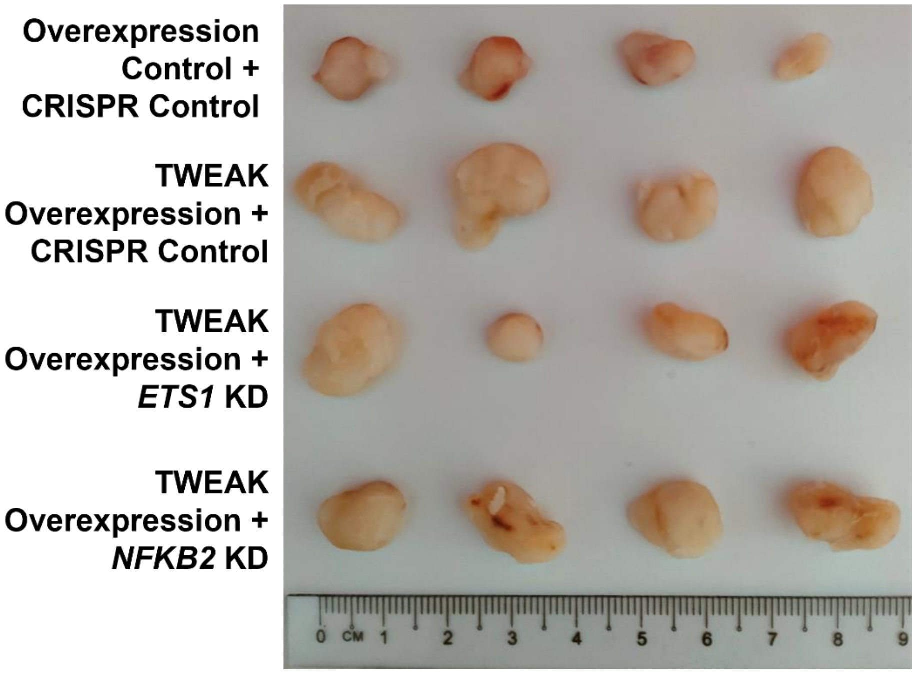

Supplementary Figure 5. Images of primary tumours extracted 35 days after mice were injected with U-87 MG cells that express either CRISPR control, *NFKB2* KD or *ETS1* KD and overexpression control or TWEAK.

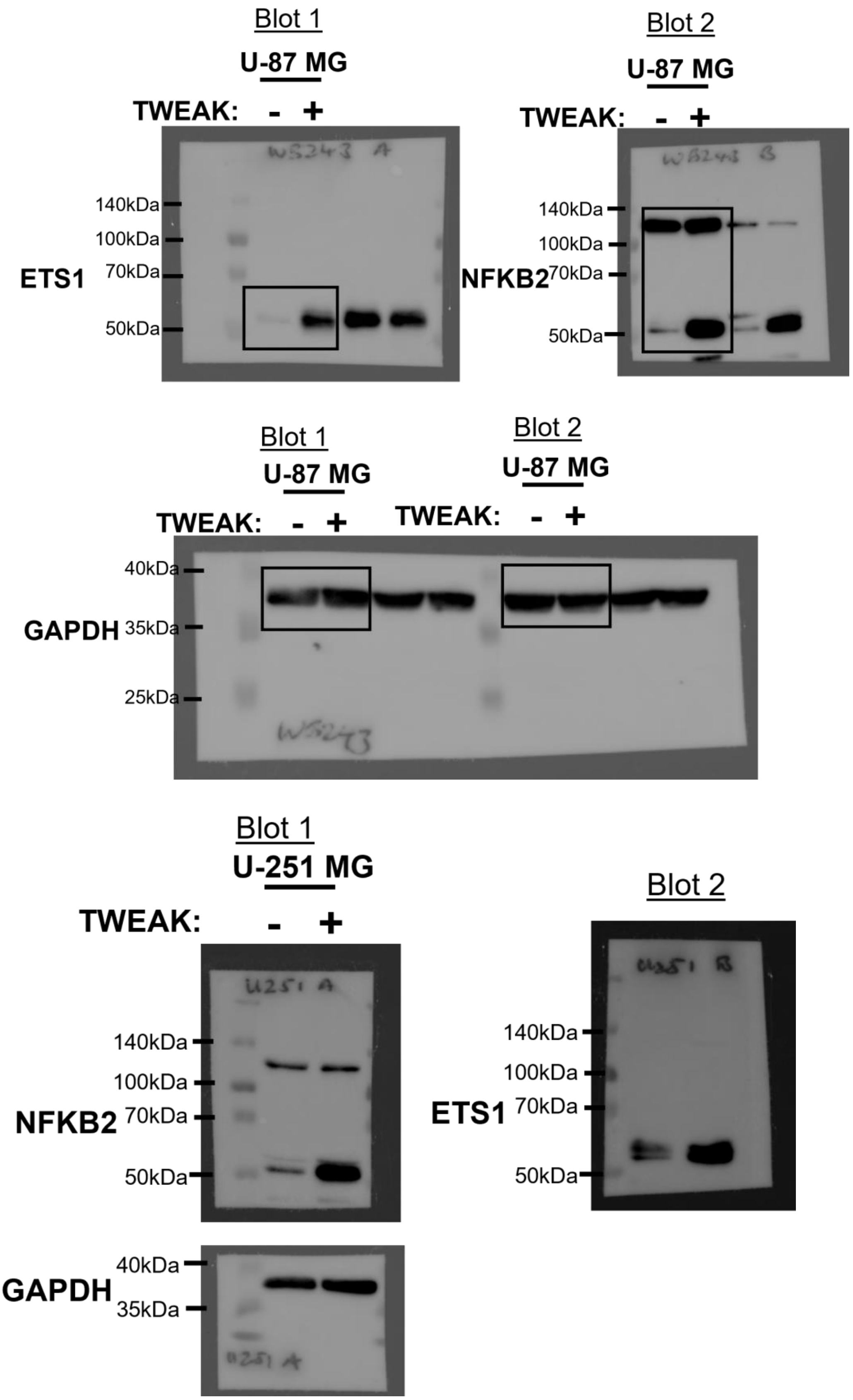

Supplementary Figure 6. Uncropped western blot images for Figure 1b. Proteins were probed on two western blots that were loaded with the same amount of protein and performed at the same time.

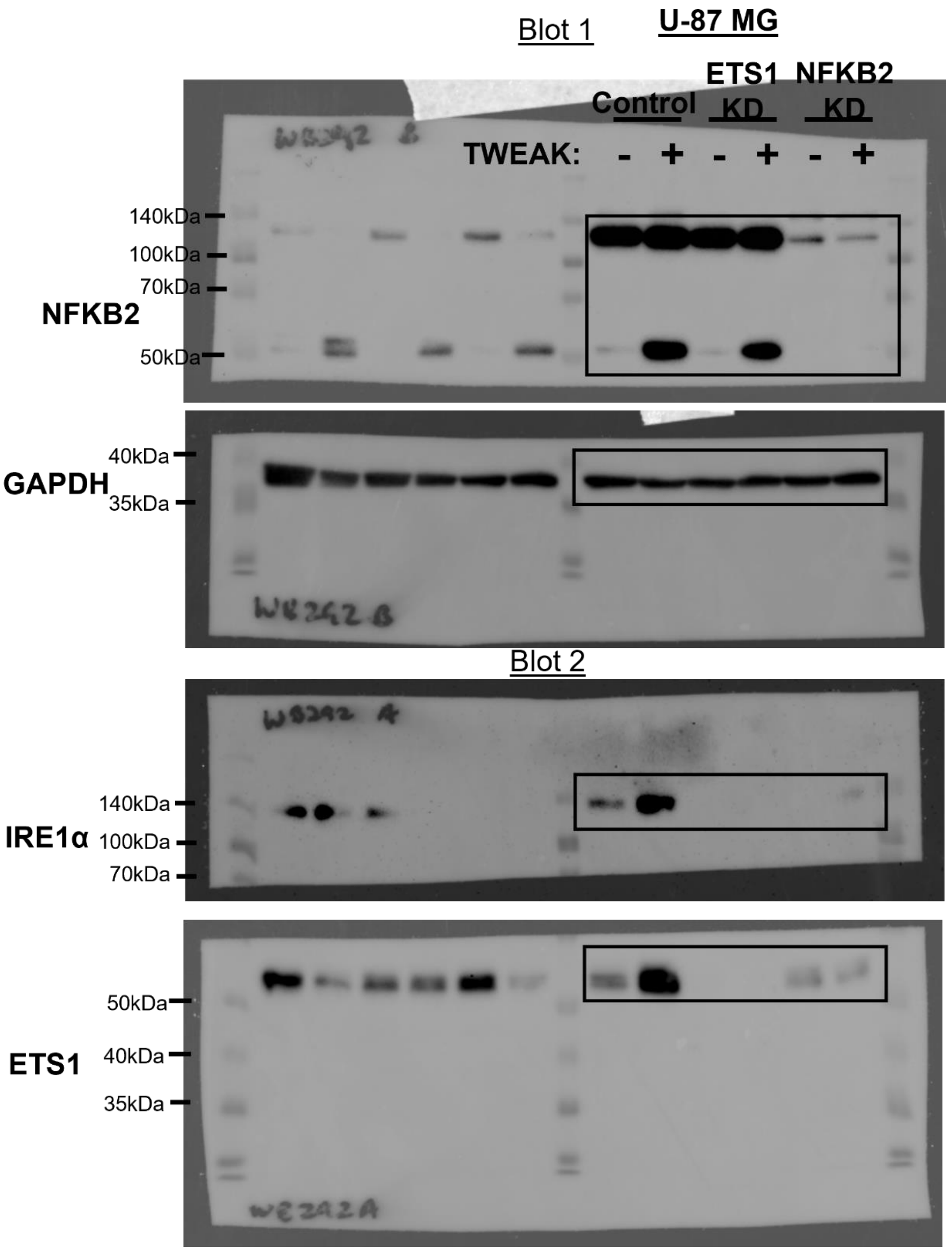

Supplementary Figure 7. Uncropped western blot images for Figure 4e U-87 MG. Proteins were probed on two western blots that were loaded with the same amount of protein and performed at the same time.

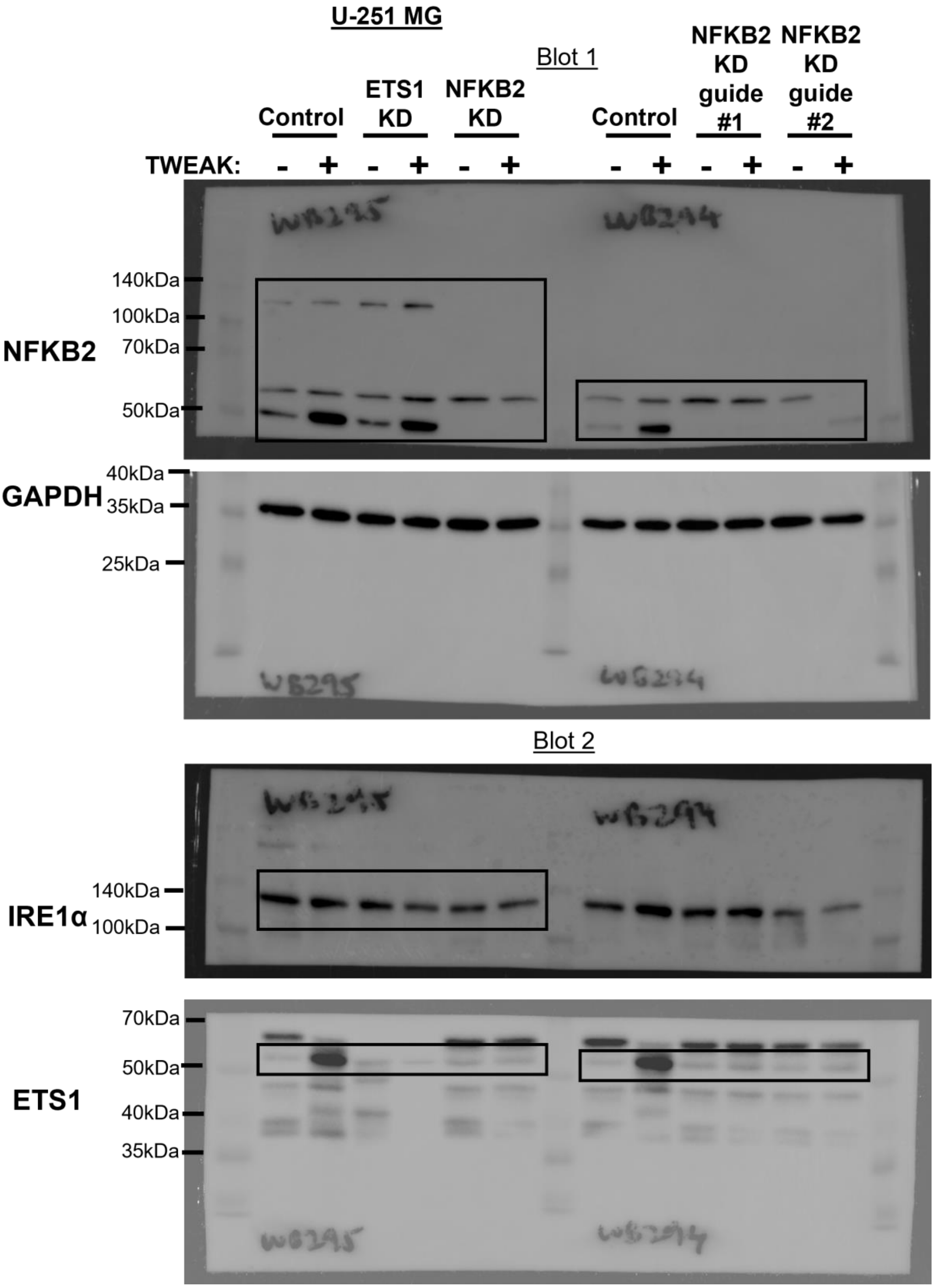

Supplementary Figure 8. Uncropped western blot images for Figure 4e and Supplementary Figure 2 U-251 MG. Proteins were probed on two western blots that were loaded with the same amount of protein and performed at the same time.

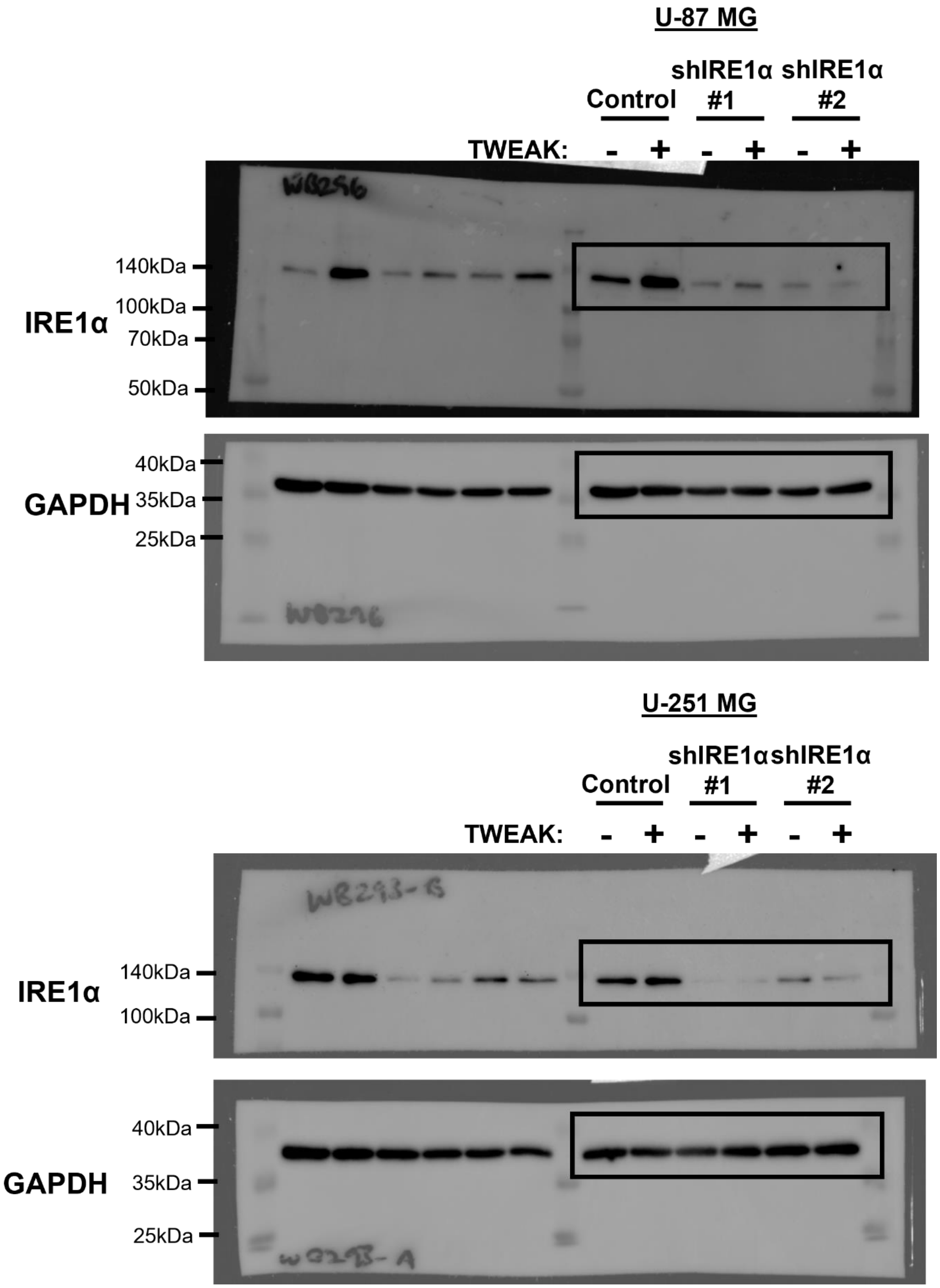

Supplementary Figure 9. Uncropped western blot images for Figure 5a. All bands were probed on the same blot.

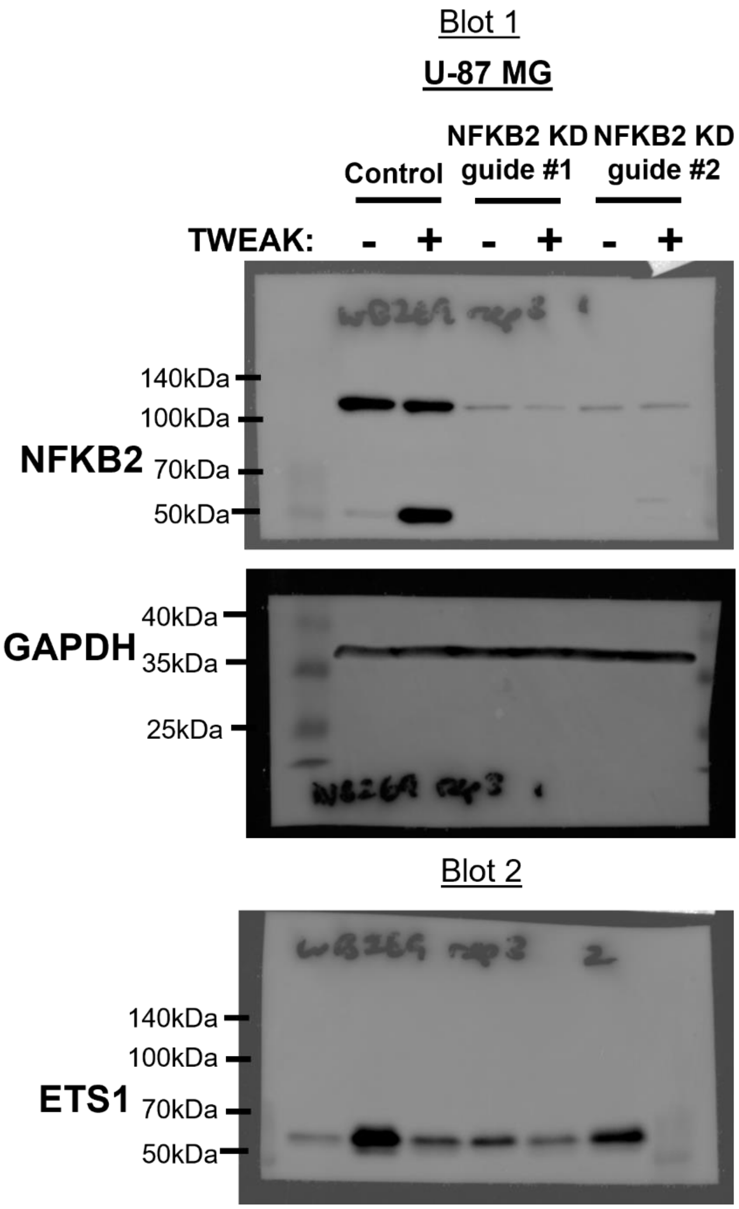

Supplementary Figure 10. Uncropped western blot images for Supplementary Figure 2 U-87 MG. Proteins were probed on two western blots that were loaded with the same amount of protein and performed at the same time.

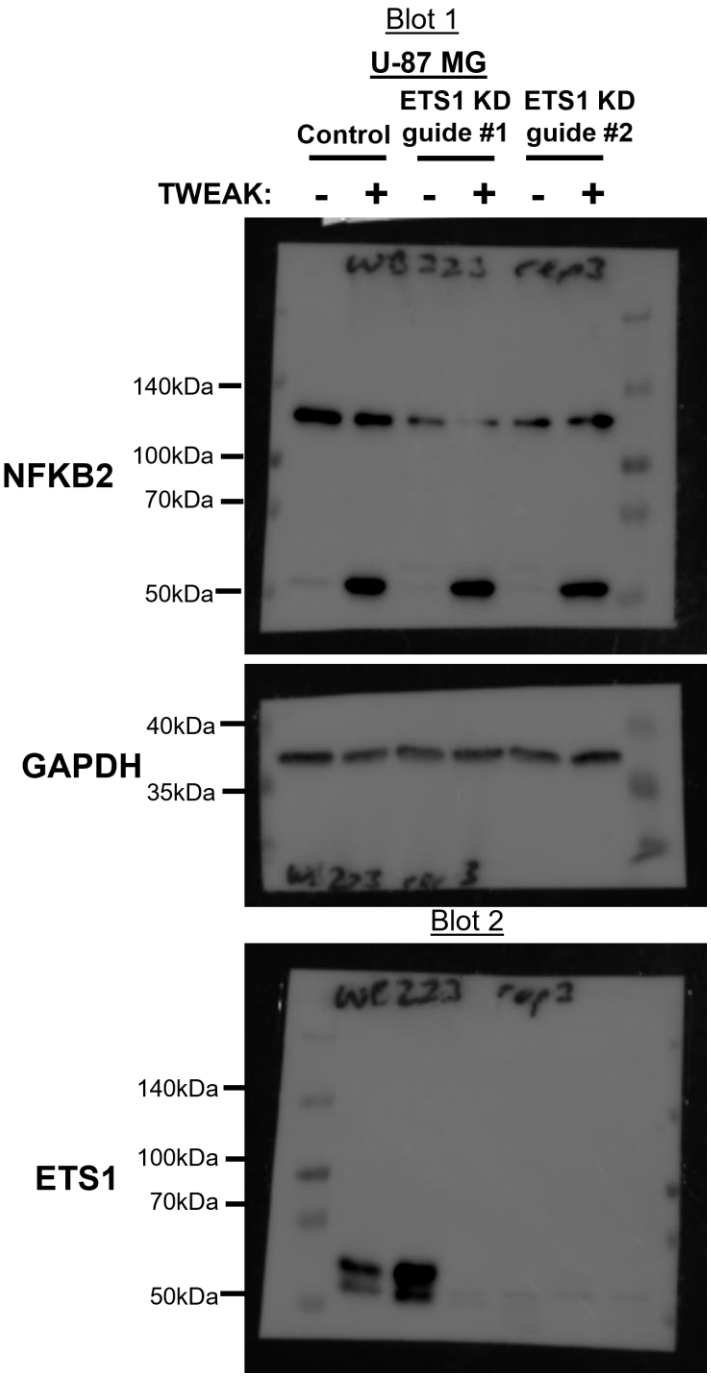

Supplementary Figure 11. Uncropped western blot images for Supplementary Figure 4 U-87 MG. Proteins were probed on two western blots that were loaded with the same amount of protein and performed at the same time.

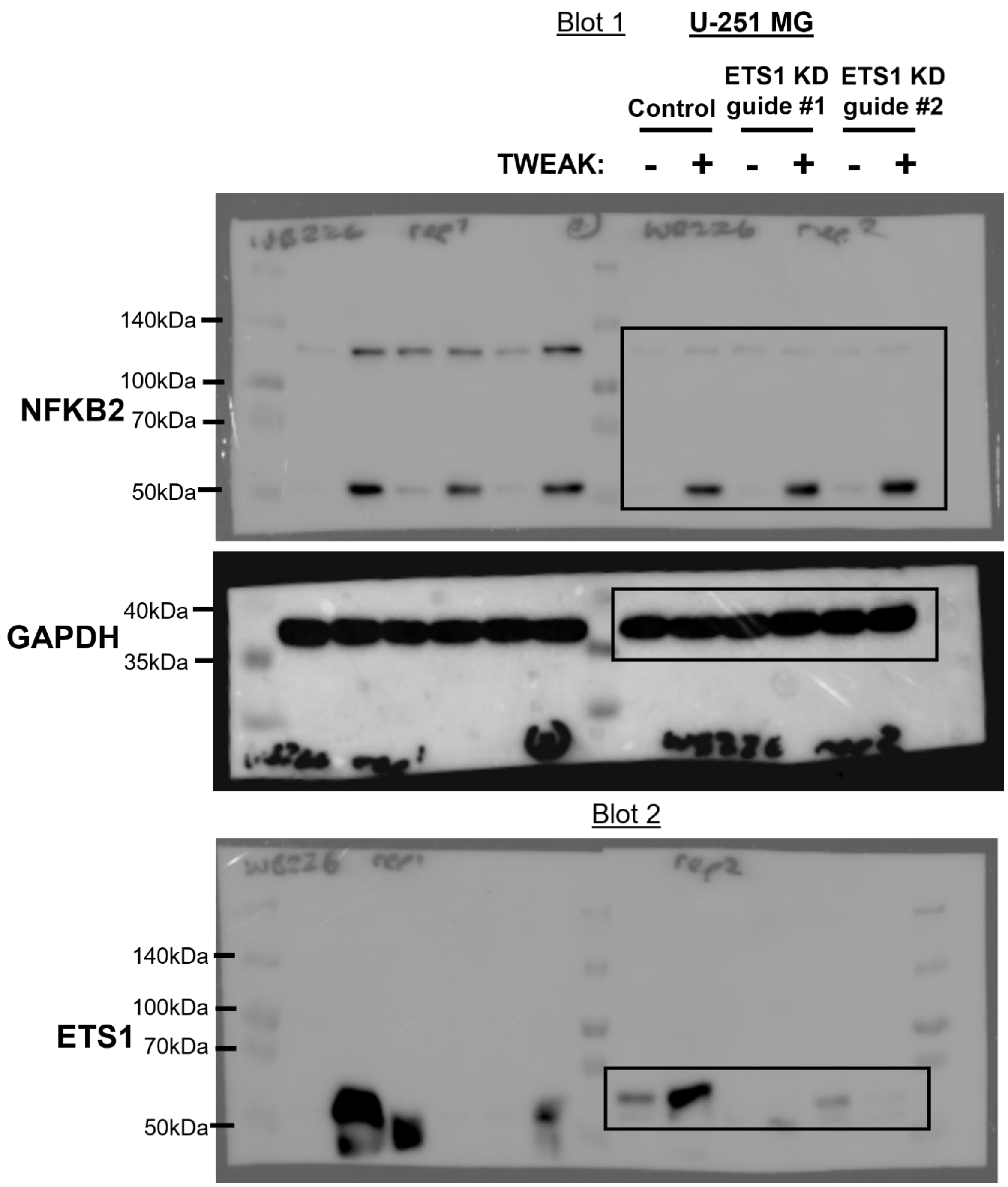

Supplementary Figure 12. Uncropped western blot images for Supplementary Figure 4 U-251 MG. Proteins were probed on two western blots that were loaded with the same amount of protein and performed at the same time.

CRISPR sgRNA sequences

Primer name	Sequence
ETS1 guide #1 F	CACCGGGTTTCTGTCCACTGCCGG
ETS1 guide #1 R	CCGGCAGTGGACAGAAACCCGGTG
ETS1 guide #2 F	CACCGCTGGGCTCTGAGAACTCCGA
ETS1 guide #2 R	AAACTCGGAGTTCTCAGAGCCCAGC
NFKB2 guide #1 F	CACCGTGGCCCCTACCTGGTGATCG
NFKB2 guide #1 R	AAACCGATCACCAGGTAGGGGCCAC
NFKB2 guide #2 F	CACCGCTTTCGGCCCTTCTCACTGG
NFKB2 guide #2 R	AAACCCAGTGAGAAGGGCCGAAAGC

shIRE1α sequences

Primer name	Sequence
shIRE1α #1	GCGTAAATTCAGGACCTATAA
shIRE1α #2	TCAACGCTGGATGGAAGTTTG

TWEAK overexpression primer sequences

Primer name	Sequence
sTWEAK F	ATACCGGTCGCCACCATGAAAACACGGGCTCGAAG
sTWEAK R	CGTATTCGAATCAGTGAACCTGGAAGAGTCCGAAGTAGGT

Supplementary Table 1. Primer sequences for CRISPR sgRNA, shIRE1α and TWEAK overexpression construct.

Genotype	Plasmid combination
CRISPR control + Overexpression control	lentiCRISPR v2 + pLJM-EGFP-hygro
CRISPR control + sTWEAK overexpression	lentiCRISPR v2 + pLJM-sTWEAK
ETS1 KD + sTWEAK overexpression	lentiCRISPR ETS1 guide #1 + pLJM-sTWEAK
NFKB2 KD + sTWEAK overexpression	lentiCRISPR NFKB2 guide #1 + pLJM-sTWEAK

Supplementary Table 2. Combinations of plasmids transduced into U-87 MG cells that were injected into immunocompromised mice and the presented genotype of the cells.
