# Supplementary material for: NF-κB/p52 augments ETS1 binding genome-wide to promote glioma progression
Source: Commun Biol. 2023 Apr 22;6:445. doi: 10.1038/s42003-023-04821-2 (PMC10122670; doi:10.1038/s42003-023-04821-2)
Supplement: Supplementary file 2 — Description of Additional Supplementary Files [file 42003_2023_4821_MOESM2_ESM.pdf]

Description of Additional Supplementary Files

File Name: Supplementary Data

Description: The source data behind Figures 1c, 2a, 2d, 3, 5b-c
