# Supplementary material for: NF-κB/p52 augments ETS1 binding genome-wide to promote glioma progression
Source: Commun Biol. 2023 Apr 22;6:445. doi: 10.1038/s42003-023-04821-2 (PMC10122670; doi:10.1038/s42003-023-04821-2)
Supplement: Supplementary file 4 — Reporting Summary [file 42003_2023_4821_MOESM4_ESM.pdf]

nature portfolio

Corresponding author(s): Li Yinghui

Last updated by author(s): Apr 3, 2023

Reporting Summary

Nature Portfolio wishes to improve the reproducibility of the work that we publish. This form provides structure for consistency and transparency in reporting. For further information on Nature Portfolio policies, see our Editorial Policies and the Editorial Policy Checklist.

Statistics

For all statistical analyses, confirm that the following items are present in the figure legend, table legend, main text, or Methods section.

n/a	Confirmed	
☐	☒	The exact sample size (*n*) for each experimental group/condition, given as a discrete number and unit of measurement
☐	☒	A statement on whether measurements were taken from distinct samples or whether the same sample was measured repeatedly
☐	☒	The statistical test(s) used AND whether they are one- or two-sided<br>*Only common tests should be described solely by name; describe more complex techniques in the Methods section.*
☒	☐	A description of all covariates tested
☒	☐	A description of any assumptions or corrections, such as tests of normality and adjustment for multiple comparisons
☐	☒	A full description of the statistical parameters including central tendency (e.g. means) or other basic estimates (e.g. regression coefficient) AND variation (e.g. standard deviation) or associated estimates of uncertainty (e.g. confidence intervals)
☐	☒	For null hypothesis testing, the test statistic (e.g. *F*, *t*, *r*) with confidence intervals, effect sizes, degrees of freedom and *P* value noted<br>*Give P values as exact values whenever suitable.*
☒	☐	For Bayesian analysis, information on the choice of priors and Markov chain Monte Carlo settings
☒	☐	For hierarchical and complex designs, identification of the appropriate level for tests and full reporting of outcomes
☒	☐	Estimates of effect sizes (e.g. Cohen's *d*, Pearson's *r*), indicating how they were calculated

Our web collection on statistics for biologists contains articles on many of the points above.

Software and code

Policy information about availability of computer code

Data collection	No Software was used
Data analysis	GraphPad Prism v8.0.1<br>R v4.1.0

For manuscripts utilizing custom algorithms or software that are central to the research but not yet described in published literature, software must be made available to editors and reviewers. We strongly encourage code deposition in a community repository (e.g. GitHub). See the Nature Portfolio guidelines for submitting code & software for further information.

Data

Policy information about availability of data

All manuscripts must include a data availability statement. This statement should provide the following information, where applicable:

- Accession codes, unique identifiers, or web links for publicly available datasets
- A description of any restrictions on data availability
- For clinical datasets or third party data, please ensure that the statement adheres to our policy

The datasets generated during and/or analysed during the current study will be made publicly available on the NCBI Gene Expression Omnibus (GEO) repository upon publication.

Human research participants

Policy information about studies involving human research participants and Sex and Gender in Research.

Reporting on sex and gender	*Use the terms sex (biological attribute) and gender (shaped by social and cultural circumstances) carefully in order to avoid confusing both terms. Indicate if findings apply to only one sex or gender; describe whether sex and gender were considered in study design whether sex and/or gender was determined based on self-reporting or assigned and methods used. Provide in the source data disaggregated sex and gender data where this information has been collected, and consent has been obtained for sharing of individual-level data; provide overall numbers in this Reporting Summary. Please state if this information has not been collected. Report sex- and gender-based analyses where performed, justify reasons for lack of sex- and gender-based analysis.*
Population characteristics	*Describe the covariate-relevant population characteristics of the human research participants (e.g. age, genotypic information, past and current diagnosis and treatment categories). If you filled out the behavioural & social sciences study design questions and have nothing to add here, write "See above."*
Recruitment	*Describe how participants were recruited. Outline any potential self-selection bias or other biases that may be present and how these are likely to impact results.*
Ethics oversight	*Identify the organization(s) that approved the study protocol.*

Note that full information on the approval of the study protocol must also be provided in the manuscript.

Field-specific reporting

Please select the one below that is the best fit for your research. If you are not sure, read the appropriate sections before making your selection.

☒ Life sciences ☐ Behavioural & social sciences ☐ Ecological, evolutionary & environmental sciences

For a reference copy of the document with all sections, see nature.com/documents/nr-reporting-summary-flat.pdf

Life sciences study design

All studies must disclose on these points even when the disclosure is negative.

Sample size	No sample size calculation was performed. We used two GBM cell lines in order to verify that results obtained from each cell line was not cell-line specific and consistent between GBM cell lines.
Data exclusions	No data was excluded from analyses
Replication	Replication were successful
Randomization	Randomization is not relevant to the current study
Blinding	Blinding is not relevant to the current study

Reporting for specific materials, systems and methods

We require information from authors about some types of materials, experimental systems and methods used in many studies. Here, indicate whether each material, system or method listed is relevant to your study. If you are not sure if a list item applies to your research, read the appropriate section before selecting a response.

Materials & experimental systems

n/a	Involved in the study	
☐	☒	Antibodies
☐	☒	Eukaryotic cell lines
☒	☐	Palaeontology and archaeology
☐	☒	Animals and other organisms
☒	☐	Clinical data
☒	☐	Dual use research of concern

Methods

n/a	Involved in the study	
☐	☒	ChIP-seq
☒	☐	Flow cytometry
☒	☐	MRI-based neuroimaging

Antibodies

Antibodies used	anti-NFkB2 (Cell Signalling; 3017)<br>anti-NFkB2 (Bethyl Laboratories; A300-BL7039)<br>anti-ETS1 (Cell Signalling; 14069)<br>anti-ETS1 (Active Motif; 39580)<br>anti-GAPDH (Santa Cruz; 32233)<br>anti-IRE1α (Santa Cruz; 390960)<br>anti-RNA polymerase II clone CTD4H8 (EMD Millipore; 05-623)
Validation	Antibody validation is available on manufacturer's website.<br>anti-NFkB2 (Cell Signalling; 3017): https://www.cellsignal.com/datasheet.jsp?productId=3017&images=1&size=A4<br>anti-ETS1 (Cell Signalling; 14069): https://www.cellsignal.com/datasheet.jsp?productId=14069&images=1&size=A4<br>anti-ETS1 (Active Motif; 39580): https://www.activemotif.com/documents/tds/39580.pdf<br>anti-GAPDH (Santa Cruz; 32233): https://datasheets.scbt.com/sc-32233.pdf<br>anti-IRE1α (Santa Cruz; 390960) : https://datasheets.scbt.com/sc-390960.pdf<br>anti-RNA polymerase II clone CTD4H8 (EMD Millipore; 05-623): https://www.merckmillipore.com/SG/en/product/Anti-RNA-polymerase-II-Antibody-clone-CTD4H8,MM_NF-05-623

Eukaryotic cell lines

Policy information about cell lines and Sex and Gender in Research

Cell line source(s)	ATCC and Sigma Alrich
Authentication	The cell lines were a gift from Phillip Koeffler at the Cancer Science Institute, Singapore and not authenticated by us.
Mycoplasma contamination	Cells were tested to be mycoplasma free using the Mycoplasma PCR Detection Kit (Applied Biological Materials Inc; G238)
Commonly misidentified lines (See ICLAC register)	U-251 MG (formerly known as U-373 MG) (ECACC 09063001)

Animals and other research organisms

Policy information about studies involving animals; ARRIVE guidelines recommended for reporting animal research, and Sex and Gender in Research

Laboratory animals	Six- to ten-week-old male Rag-/- IL2γ-/- mice were used in all experiments.
Wild animals	The study did not involve wild animals.
Reporting on sex	Only male mice were used.
Field-collected samples	The study did not involve samples collected from the field.
Ethics oversight	All animal experiments were performed in compliance with protocols approved by the Nanyang Technological University Institutional Animal Care and Use Committee (IACUC)

Note that full information on the approval of the study protocol must also be provided in the manuscript.

ChIP-seq

Data deposition

☒ Confirm that both raw and final processed data have been deposited in a public database such as GEO.

☒ Confirm that you have deposited or provided access to graph files (e.g. BED files) for the called peaks.

Data access links<br>*May remain private before publication.*	https://www.ncbi.nlm.nih.gov/geo/query/acc.cgi?acc=GSE207982<br>Reviewer access token: uxkfqqcihjevnep
Files in database submission	U87 Control UT p52 (merged bigwig): U87_C_UT_p52_sorted_merged.bw<br>U87 Control TWEAK p52 (merged bigwig): U87_C_p52_sorted_merged.bw<br>U87 Control UT ETS1 (merged bigwig): U87_C_UT_ETS_sorted_merged.bw<br>U87 Control TWEAK ETS1 (merged bigwig): U87_C_TW_ETS_sorted_merged.bw<br>U87 ETS1 KD guide 1 TWEAK p52 (merged bigwig): U87_Ex5_p52_sorted_merged.bw<br>U87 ETS1 KD guide 2 TWEAK p52 (merged bigwig): U87_Ex7_p52_sorted_merged.bw<br>U87 Control UT RNApol2 (merged bigwig): U87_C_UT_RNApol2_merged.bw<br>U87 Control TWEAK RNApol2 (merged bigwig): U87_C_TW_RNApol2_merged.bw<br>U87 ETS1KD TWEAK RNApol2 (merged bigwig): U87_ETS1KD_TW_RNApol2_merged.bw<br>U87 NFKB2KD TWEAK RNApol2 (merged bigwig): U87_NFKB2KD_TW_RNApol2_merged.bw

U87 control input raw file: U87_C_TW1_input_DKDL220001442-1a-18_HHKVYCCX2_L7_1.fq.gz
U87_C_TW1_input_DKDL220001442-1a-18_HHKVYCCX2_L7_2.fq.gz
U87 ETS1 KD guide 1 input raw file: U87_Ex5_TW_input_DKDL220003595-1a-29_HHF7WCCX2_L7_1.fq.gz
U87_Ex5_TW_input_DKDL220003595-1a-29_HHF7WCCX2_L7_2.fq.gz
U87 ETS1 KD guide 2 input raw file: U87_Ex7_TW_input_DKDL220003595-1a-31_HHF7WCCX2_L7_1.fq.gz
U87_Ex7_TW_input_DKDL220003595-1a-31_HHF7WCCX2_L7_2.fq.gz
U87 Control UT p52 rep 1 raw file: U87_C_UT_p52_rep1_DKDL220003596-1a-32_HHF7WCCX2_L7_1.fq.gz
87_C_UT_p52_rep1_DKDL220003596-1a-32_HHF7WCCX2_L7_2.fq.gz
U87 Control UT p52 rep 2 raw file: U87_C_UT_p52_rep2_DKDL220003596-1a-33_HHF7WCCX2_L7_1.fq.gz
U87 Control TWEAK p52 rep 1 raw file: U87_C_p52_TW1_DKDL220001442-1a-19_HHKVYCCX2_L7_1.fq.gz
U87_C_p52_TW1_DKDL220001442-1a-19_HHKVYCCX2_L7_2.fq.gz
U87 Control TWEAK p52 rep 2 raw file: U87_C_p52_TW2_DKDL220001442-1a-20_HHKVYCCX2_L7_1.fq.gz
U87_C_p52_TW2_DKDL220001442-1a-20_HHKVYCCX2_L7_2.fq.gz
U87 Control UT ETS1 rep1 raw file: U87_C_UT_ETS_rep1_DKDL220003596-1a-34_HHF7WCCX2_L7_1.fq.gz
U87_C_UT_ETS_rep1_DKDL220003596-1a-34_HHF7WCCX2_L7_2.fq.gz
U87 Control UT ETS1 rep2 raw file: U87_C_UT_ETS_rep2_DKDL220003596-1a-35_HHF7WCCX2_L7_1.fq.gz
U87_C_UT_ETS_rep2_DKDL220003596-1a-35_HHF7WCCX2_L7_2.fq.gz
U87 Control TWEAK ETS1 rep1 raw file: U87_C_TW_ETS_rep1_DKDL220003596-1a-36_HHF7WCCX2_L7_1.fq.gz
U87_C_TW_ETS_rep1_DKDL220003596-1a-36_HHF7WCCX2_L7_2.fq.gz
U87 Control TWEAK ETS1 rep2 raw file: U87_C_TW_ETS_rep2_DKDL220003596-1a-42_HHF7WCCX2_L7_1.fq.gz
U87_C_TW_ETS_rep2_DKDL220003596-1a-42_HHF7WCCX2_L7_2.fq.gz
U87 ETS1 KD guide 1 TWEAK p52 rep 1: U87_Ex5_p52_TW1_DKDL220001442-1a-21_HHKVYCCX2_L7_1.fq.gz
U87_Ex5_p52_TW1_DKDL220001442-1a-21_HHKVYCCX2_L7_2.fq.gz
U87 ETS1 KD guide 1 TWEAK p52 rep 2: U87_Ex5_p52_TW2_DKDL220001442-1a-22_HHKVYCCX2_L7_1.fq.gz
U87_Ex5_p52_TW2_DKDL220001442-1a-22_HHKVYCCX2_L7_2.fq.gz
U87 ETS1 KD guide 2 TWEAK p52 rep 1: U87_Ex7_p52_TW1_DKDL220001442-1a-23_HHKVYCCX2_L7_1.fq.gz
U87_Ex7_p52_TW1_DKDL220001442-1a-23_HHKVYCCX2_L7_2.fq.gz
U87 ETS1 KD guide 2 TWEAK p52 rep 2: U87_Ex7_p52_TW2_DKDL220001442-1a-25_HHKVYCCX2_L7_1.fq.gz
U87_Ex7_p52_TW2_DKDL220001442-1a-25_HHKVYCCX2_L7_2.fq.gz
U87 Control UT RNApol2 raw file rep1: U87_C_UT1_DKDL220012605-1A_HJJ2VCCX2_L7_1.fq.gz
U87_C_UT1_DKDL220012605-1A_HJJ2VCCX2_L7_2.fq.gz
U87 Control UT RNApol2 raw file rep2: U87_C_UT2_DKDL220012605-1A_HJJ2VCCX2_L7_1.fq.gz
U87_C_UT2_DKDL220012605-1A_HJJ2VCCX2_L7_2.fq.gz
U87 Control TWEAK RNApol2 raw file rep1: U87_C_TW1_DKDL220012605-1A_HJJ2VCCX2_L7_1.fq.gz
U87_C_TW1_DKDL220012605-1A_HJJ2VCCX2_L7_2.fq.gz
U87 Control TWEAK RNApol2 raw file rep2: U87_C_TW2_DKDL220012605-1A_HJJ2VCCX2_L7_1.fq.gz
U87_C_TW2_DKDL220012605-1A_HJJ2VCCX2_L7_2.fq.gz
U87 ETS1 KD TWEAK RNApol2 raw file rep1: U87_ETS1KD_TW1_DKDL220012605-1A_HJJ2VCCX2_L7_1.fq.gz
U87_ETS1KD_TW1_DKDL220012605-1A_HJJ2VCCX2_L7_2.fq.gz
U87 ETS1 KD TWEAK RNApol2 raw file rep2: U87_ETS1KD_TW2_DKDL220012605-1A_HJJ2VCCX2_L7_1.fq.gz
U87_ETS1KD_TW2_DKDL220012605-1A_HJJ2VCCX2_L7_2.fq.gz
U87 NFKB2 KD TWEAK RNApol2 raw file rep1: U87_p52KD_TW1_DKDL220012605-1A_HJJ2VCCX2_L7_1.fq.gz
U87_p52KD_TW1_DKDL220012605-1A_HJJ2VCCX2_L7_2.fq.gz
U87 NFKB2 KD TWEAK RNApol2 raw file rep2: U87_p52KD_TW2_DKDL220012605-1A_HJJ2VCCX2_L7_1.fq.gz
U87_p52KD_TW2_DKDL220012605-1A_HJJ2VCCX2_L7_2.fq.gz
U87 Control UT RNAseq raw file rep 1: U87_C_UT1_DKDL220012617-1A_HJJF2CCX2_L6_1.fq.gz
U87_C_UT1_DKDL220012617-1A_HJJF2CCX2_L6_2.fq.gz
U87 Control UT RNAseq raw file rep 2: U87_C_UT2_DKDL220012617-1A_HJJF2CCX2_L6_1.fq.gz
U87_C_UT2_DKDL220012617-1A_HJJF2CCX2_L6_2.fq.gz
U87 Control UT RNAseq raw file rep 3: U87_C_UT3_DKDL220012617-1A_HJJF2CCX2_L6_1.fq.gz
U87_C_UT3_DKDL220012617-1A_HJJF2CCX2_L6_2.fq.gz
U87 Control TWEAK RNAseq raw file rep 1: U87_C_TW1_DKDL220012617-1A_HJJF2CCX2_L6_1.fq.gz
U87_C_TW1_DKDL220012617-1A_HJJF2CCX2_L6_2.fq.gz
U87 Control TWEAK RNAseq raw file rep 2: U87_C_TW2_DKDL220012617-1A_HJJF2CCX2_L6_1.fq.gz
U87_C_TW2_DKDL220012617-1A_HJJF2CCX2_L6_2.fq.gz
U87 Control TWEAK RNAseq raw file rep 3: U87_C_TW3_DKDL220012617-1A_HJJF2CCX2_L6_1.fq.gz
U87_C_TW3_DKDL220012617-1A_HJJF2CCX2_L6_2.fq.gz
U87 ETS1 KD TWEAK RNAseq raw file rep 1: U87_ETS1KD_TW1_DKDL220012617-1A_HJJF2CCX2_L6_1.fq.gz
U87_ETS1KD_TW1_DKDL220012617-1A_HJJF2CCX2_L6_2.fq.gz
U87 ETS1 KD TWEAK RNAseq raw file rep 2: U87_ETS1KD_TW2_DKDL220012617-1A_HJJF2CCX2_L6_1.fq.gz
U87_ETS1KD_TW2_DKDL220012617-1A_HJJF2CCX2_L6_2.fq.gz
U87 ETS1 KD TWEAK RNAseq raw file rep 3: U87_ETS1KD_TW3_DKDL220012617-1A_HJJF2CCX2_L6_1.fq.gz
U87_ETS1KD_TW3_DKDL220012617-1A_HJJF2CCX2_L6_2.fq.gz
U87 NFKB2 KD TWEAK RNAseq raw file rep 1: U87_p52KD_TW1_DKDL220012617-1A_HJJF2CCX2_L6_1.fq.gz
U87_p52KD_TW1_DKDL220012617-1A_HJJF2CCX2_L6_2.fq.gz
U87 NFKB2 KD TWEAK RNAseq raw file rep 2: U87_p52KD_TW2_DKDL220012617-1A_HJJF2CCX2_L6_1.fq.gz
U87_p52KD_TW2_DKDL220012617-1A_HJJF2CCX2_L6_2.fq.gz
U87 NFKB2 KD TWEAK RNAseq raw file rep 3: U87_p52KD_TW3_DKDL220012617-1A_HJJF2CCX2_L6_1.fq.gz
U87_p52KD_TW3_DKDL220012617-1A_HJJF2CCX2_L6_2.fq.gz

U87 Control UT p52 rep1 narrowpeaks: U87_C_UT_p52_rep1_peaks.narrowPeak
U87 Control UT p52 rep2 narrowpeaks: U87_C_UT_p52_rep2_peaks.narrowPeak
U87 Control TW p52 rep1 narrowpeaks: U87_C_p52_TW1_peaks.narrowPeak
U87 Control TW p52 rep2 narrowpeaks: U87_C_p52_TW2_peaks.narrowPeak

U87 Control UT ETS1 rep1 narrowpeaks: U87_C_UT_ETS_rep1_peaks.narrowPeak
U87 Control UT ETS1 rep2 narrowpeaks: U87_C_UT_ETS_rep2_peaks.narrowPeak
U87 Control TW ETS1 rep1 narrowpeaks: U87_C_TW_ETS_rep1_peaks.narrowPeak
U87 Control TW ETS1 rep2 narrowpeaks: U87_C_TW_ETS_rep2_peaks.narrowPeak
U87 ETS1 KD guide 1 TWEAK p52 rep 1 narrowpeaks:U87_Ex5_p52_TW1_peaks.narrowPeak
U87 ETS1 KD guide 1 TWEAK p52 rep 2 narrowpeaks:U87_Ex5_p52_TW2_peaks.narrowPeak
U87 ETS1 KD guide 2 TWEAK p52 rep 1 narrowpeaks:U87_Ex7_p52_TW1_peaks.narrowPeak
U87 ETS1 KD guide 2 TWEAK p52 rep 2 narrowpeaks:U87_Ex7_p52_TW2_peaks.narrowPeak
U87 Control UT RNApol2 rep1 narrowpeaks: U87_C_UT1_peaks.narrowPeak
U87 Control UT RNApol2 rep2 narrowpeaks: U87_C_UT2_peaks.narrowPeak
U87 Control TWEAK RNApol2 rep1 narrowpeaks: U87_C_TW1_peaks.narrowPeak
U87 Control TWEAK RNApol2 rep2 narrowpeaks: U87_C_TW2_peaks.narrowPeak
U87 ETS1 KD TWEAK RNApol2 rep1 narrowpeaks: U87_ETS1KD_TW1_peaks.narrowPeak
U87 ETS1 KD TWEAK RNApol2 rep2 narrowpeaks: U87_ETS1KD_TW2_peaks.narrowPeak
U87 NFKB2 KD TWEAK RNApol2 rep1 narrowpeaks: U87_p52KD_TW1_peaks.narrowPeak
U87 NFKB2 KD TWEAK RNApol2 rep2 narrowpeaks: U87_p52KD_TW2_peaks.narrowPeak
U87 RNA-seq raw counts: U87_RNAseq_counts.bed

Genome browser session
(e.g. UCSC)

https://genome.ucsc.edu/s/Nicholas.Sim/U87ChIP

Methodology

Replicates

All ChIP Seq experiments were done in Biological Duplicate.

Sequencing depth

All sequencing were done to 150bp paired-end.

Antibodies

anti-NFkB2 (Bethyl Laboratories; A300-BL7039)
anti-ETS1 (Active Motif; 39580)
anti-RNA polymerase II clone CTD4H8 (EMD Millipore; 05-623)

Peak calling parameters

Peaks were called using MACS2 with subtraction of input signal. Blacklisted regions were removed from the called peaks using bedtools

Data quality

The quality of sequencing reads were assessed using FastQC.
Trimmomatic was used to remove adaptors, and reassessed with FastQC to ensure all adaptor sequences were removed.
ChIP-seq reads were mapped to hg38 using Bowtie2.
Peaks were called using MACS2 with subtraction of input signal.
Blacklisted regions were removed from the called peaks using bedtools.

Number of peaks called on MACS2 at FDR 0.1:
U87 Control UT p52 rep1: 226
U87 Control UT p52 rep2: 120
U87 Control TW p52 rep1: 2979
U87 Control TW p52 rep2: 11320
U87 Control UT ETS1 rep1: 3424
U87 Control UT ETS1 rep2: 1497
U87 Control TW ETS1 rep1: 2975
U87 Control TW ETS1 rep2: 2524
U87 ETS1 KD guide 1 TWEAK p52 rep 1: 1207
U87 ETS1 KD guide 1 TWEAK p52 rep 2: 2622
U87 ETS1 KD guide 2 TWEAK p52 rep 1: 3963
U87 ETS1 KD guide 2 TWEAK p52 rep 2: 3582
U87 Control UT RNA pol2 rep1: 24848
U87 Control UT RNA pol2 rep2: 38307
U87 Control TWEAK RNA pol2 rep1: 44672
U87 Control TWEAK RNA pol2 rep2: 27056
U87 ETS1 KD TWEAK RNA pol2 rep1: 24710
U87 ETS1 KD TWEAK RNA pol2 rep2: 29785
U87 NFKB2 KD TWEAK RNA pol2 rep1: 30310
U87 NFKB2 KD TWEAK RNA pol2 rep2: 11175

Software

Assessment of sequencing read quality: FastQC.
Removal of adaptor sequences: Trimmomatic.
Mapping reads to hg38 for ChIP-seq: Bowtie2
Peak calling: MACS2
Removal of peaks: bedtools
Generation of heatmaps: deepTools
Motif analysis: HOMER and MEME-ChIP
Differential analysis for RNA pol2 ChIP: diffBind
